# Single-base resolution quantitative genome methylation analysis in the model bacterium *Helicobacter pylori* by enzymatic methyl sequencing (EM-Seq) reveals influence of strain, growth phase, and methyl homeostasis

**DOI:** 10.1186/s12915-024-01921-1

**Published:** 2024-05-29

**Authors:** Lubna Patel, Florent Ailloud, Sebastian Suerbaum, Christine Josenhans

**Affiliations:** Max von Pettenkofer Institute, Chair for Medical Microbiology, Faculty of Medicine, LMU Munich, Pettenkoferstr. 9a, 80336 Munich, Germany

**Keywords:** Epigenetics, Methylome, High-throughput sequencing, DNA methyltransferases, Bacteria, Epigenetic changes, *Helicobacter pylori*

## Abstract

**Background:**

Bacterial epigenetics is a rapidly expanding research field. DNA methylation by diverse bacterial methyltransferases (MTases) contributes to genomic integrity and replication, and many recent studies extended MTase function also to global transcript regulation and phenotypic variation. *Helicobacter pylori* is currently one of those bacterial species which possess the highest number and the most variably expressed set of DNA MTases. Next-generation sequencing technologies can directly detect DNA base methylation. However, they still have limitations in their quantitative and qualitative performance, in particular for cytosine methylation.

**Results:**

As a complementing approach, we used enzymatic methyl sequencing (EM-Seq), a technology recently established that has not yet been fully evaluated for bacteria. Thereby, we assessed quantitatively, at single-base resolution, whole genome cytosine methylation for all methylated cytosine motifs in two different *H. pylori* strains and isogenic MTase mutants. EM-Seq reliably detected both ^m5^C and ^m4^C methylation. We demonstrated that three different active cytosine MTases in *H. pylori* provide considerably different levels of average genome-wide single-base methylation, in contrast to isogenic mutants which completely lost specific motif methylation. We found that strain identity and changed environmental conditions, such as growth phase and interference with methyl donor homeostasis, significantly influenced quantitative global and local genome-wide methylation in *H. pylori* at specific motifs. We also identified significantly hyper- or hypo-methylated cytosines, partially linked to overlapping MTase target motifs. Notably, we revealed differentially methylated cytosines in genome-wide coding regions under conditions of methionine depletion, which can be linked to transcript regulation.

**Conclusions:**

This study offers new knowledge on *H. pylori* global and local genome-wide methylation and establishes EM-Seq for quantitative single-site resolution analyses of bacterial cytosine methylation.

**Supplementary Information:**

The online version contains supplementary material available at 10.1186/s12915-024-01921-1.

## Background

Epigenetic modifications of nucleic acids such as DNA and RNA, for instance by methylation, play a major role in regulating cellular processes in all kingdoms of life. Modern high-throughput methods have greatly helped to understand epigenetic modifications at a larger, genome-wide scale and to identify rare modifications. In particular, third-generation sequencing technologies have identified numerous previously unknown modifications in diverse organisms [1]. Although known for a long time, the precise and diverse biological functions of DNA methylation and methyl transferases (MTases) in bacteria, whether part of restriction-modification systems or as orphan genes, are still understudied. Restriction-modification [methylation] systems have been widely characterized as “immune systems” of bacteria, which prevent or minimize the entry of heterologous DNA, in particular by bacteriophage infection [2], and also contribute to DNA integrity and replication [3]. Comprehensive bacterial epigenetics is a quite recently expanded field, which has shown that numerous bacterial species express multiple MTases, which methylate DNA at specific nucleotide recognition motifs that are distributed throughout the chromosome [1]. Dam methylase (performing N6-adenine methylation at GATC motifs) has been intensively characterized to play an essential role for DNA integrity, mutation protection, and replication [3, 4]. In addition, many recent studies expanded the function of bacterial MTases, including Dam MTase, for instance to global transcript regulation and phenotype bistability [5–9].

The bacterial human stomach pathogen, *Helicobacter pylori*, is one of the bacterial species with the highest number and the most variable and variably expressed portfolio of DNA MTases [10–15]. *H. pylori* strains express highly variable sets of ~ 20 active MTases of different types [11–13]. This set also includes a number of orphan MTases, which are not accompanied by a cognate restriction endonuclease [16]. Three highly conserved MTases in *H. pylori* have been shown to be crucially involved in global transcriptional regulation [16–18], partially in a strain-specific manner. MTase involvement in global regulation has also been demonstrated in other bacteria [7, 19, 20]. For *H. pylori*, MTase activity and motif-specific genome methylation are highly variable. The presence and activity of the respective enzymes are partially strain- and phase-variable [13, 21]; the target specificity of some MTases can also be variable due to frameshifting, generating active enzymes of different length and target preference [13]. In addition, the compendium of MTases in *H. pylori* varies between clinical isolates and is under continuous evolution in vivo in human patients, probably benefiting the persistently infecting lifestyle of the bacteria [15, 22]. So far, MTase activity on specific target motifs over the whole genome of *H. pylori* has been detected using PacBio Single-Molecule, Real-Time (SMRT) sequencing or, more recently, Oxford Nanopore sequencing technology (ONT) [13, 23–25]. Sequencing-based methodology allows unsurpassed detail in genome-wide analysis by the direct detection of modified bases during sequencing. Although third-generation sequencing-based methodology is highly performing and offers single-nucleotide resolution under specific conditions, it is rather cost-intensive, and bioinformatics for reliable detection of nucleotide modifications require large prior training sets to be built and analyzed. In particular, ^m5^C methylation is less reliably detected directly from PacBio sequencing reads, due to low signal strength with untreated DNA [13, 26]. Previously established, sequencing-independent methods such as dot blots do not directly detect site-specific methylation, are much less sensitive, and do not provide single-nucleotide resolution quantitative information on each motif-specific genome-wide nucleotide methylation [12, 27, 28].

Investigating bacterial DNA MTases and their DNA targets is interesting in various contexts. First, the variety, conservation and fundamental biological functions of these important enzymes are still understudied. Quantitative information about the local methylation frequency at target motifs of any *H. pylori* MTase within an individual bacterial genome at any given time point and condition is lacking. The term “local quantitative methylation at single-base resolution” designates the percentage at which each specific methylatable cytosine position is actually methylated in a bacterial population, as opposed to analyses of global methylation detection, i.e., whether a specific DNA motif can be methylated by a given MTase. In addition, the biological roles of the MTases, whether occurring in combination with restriction enzymes, or as orphan MTases [22], are still largely unknown. Moreover, the inhibition of methylation may represent an interesting new strategy to antagonize and eliminate the bacteria from an infection or other pathogenic setting. For all those purposes, it is important to establish easy and practical, high-throughput methods which help to analyze qualitative, quantitative, and if possible, localized, aspects of the methylation events and outcomes under various conditions.

In the present study, we have applied a recently introduced high-throughput genome-wide analysis method to detect bacterial DNA methylation at cytosines, enzymatic methyl sequencing (EM-Seq), to the model bacterium *H. pylori* [29]. This method is based on the enzymatic conversion of non-methylated cytosines to thymines, combined with Illumina sequencing, and has been found highly performing on eukaryotic DNA for ^m5^C methylation, even at the single-base level [29, 30]. We now used this highly quantitative approach to characterize local epigenetic cytosine modifications in *H. pylori* wild type strains, selected MTase mutants, and *luxS* mutants, also under various culture conditions. We found that different active MTase enzymes methylate their cognate motifs, both at ^m5^C and ^m4^C, to a different global and local quantitative extent. We also underscore that different mutations, growth phases and environmental conditions influence the global and local methylation patterns quantitatively and differentially. Local analyses of methylation at single-base resolution reveal intriguing sites of hypo-methylation and differential methylation, in particular under conditions of methionine depletion.

## Results

### Detecting comprehensive bacterial genome methylation in *H. pylori*

**Fig. 1:**
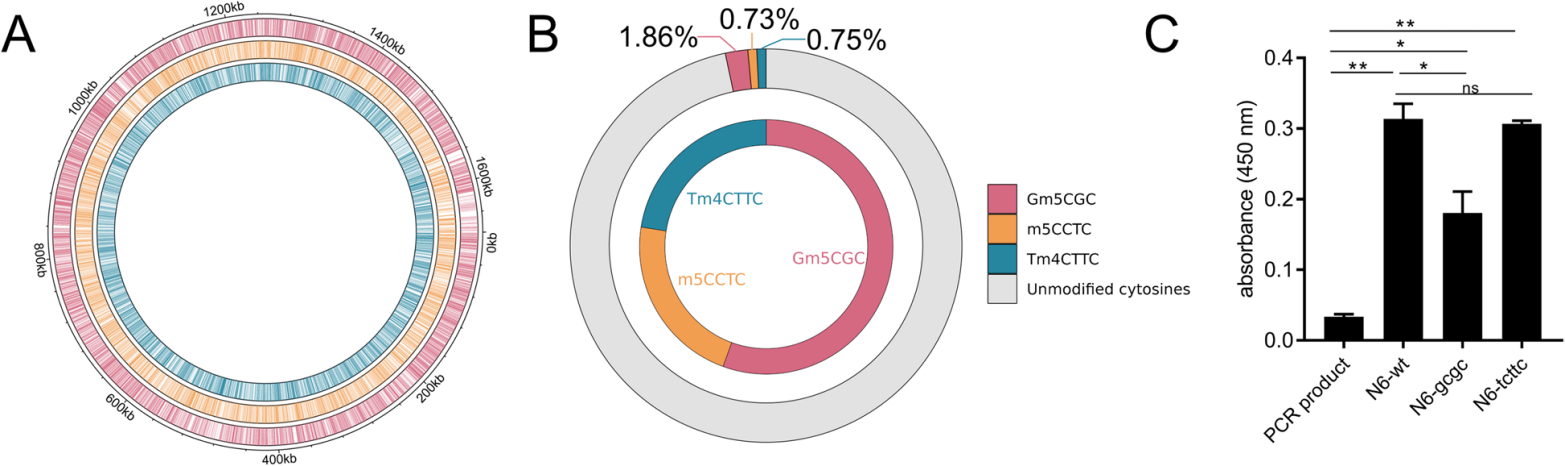
Broadly predicting and detecting DNA cytosine methylation in the *H. pylori* genome.** A** Predicted distribution of GCGC (red), CCTC (orange), and TCTTC (petrol) methylation motifs along the genome of *H. pylori* strain N6. **B** Proportion (percentage) of potentially methylated cytosines at the GCGC (red label), CCTC (orange label), and TCTTC (petrol label) motifs among all cytosines in the *H. pylori* N6 genome (outer circle). Proportion of each type of cytosine-methylatable motif among all methylated cytosines in the genome of *H. pylori N6* (inner circle). **C** Experimental bulk detection of methylated cytosines in *H. pylori* N6 wild type strain (N6-wt) and MTase (G^m5^CGC MTase – N6-gcgc, M.HpyAVIII; T^m4^CTTC MTase – N6-tcttc, M2.HpyAII) isogenic mutants, by ^m5^C-specific antibody ELISA in multi-well plates. PCR product is shown as non-methylated negative control. ^m4^C methylation in TCTTC motif (N6-tcttc) is not recognized by the.^m5^C-specific antibody. Statistically significant differences between conditions were calculated using ordinary two-way ANOVA. Significances are marked: *p < 0.05; **p < 0.01

*H. pylori* is a paradigm organism for the study of epigenetic modification in bacteria, since it possesses an exceptional wealth of diverse DNA-methylating enzymes and cognate target sequences. Each strain expresses a set of approximately 20 active MTases, capable of catalyzing ^m5^C, ^m4^C, or ^m6^A DNA modifications [13, 16, 27]. Strain-specific sets of MTases in *H. pylori* are highly diverse [13, 18, 22]. Genome methylation in *H. pylori* has previously been primarily detected by restriction enzyme protection assays and antibody-based detection assays in bulk format, such as dot blots using custom antibodies [10, 11, 27], which do not offer high sensitivity or resolution. “Third-generation” long-read sequencing analyses (PacBio) [31] have been used to detect genome-wide methylation in *H. pylori* [13] despite limitations with respect to the detection of cytosine (^m5^C) methylation and quantitation at a single-base resolution. This study now evaluates a method for methylation analysis that is complementary to third-generation sequencing in both these respects. In Fig. 1A, we show the genome-wide distribution of three MTase target motifs leading to DNA cytosine methylation for *H. pylori* strain N6, which were detected before by SMRT sequencing for strains 26695, J99 [13], and BCM-300 [13, 23]. Calculating the expected methylation at certain cytosine motifs (Fig. 1B, C), we also found that at best about 3% of all cytosines in the genome, situated in the known specific target motifs, are predicted to be methylated (see also [22]), which does not afford a sufficiently high sensitivity to quantitate minor methylation changes with any bulk detection methodology. The GCGC motif is calculated to be the most prevalent of the predicted methylated motifs in *H. pylori* strain N6 and in other strains, at about 1.8% of all cytosines (Fig. 1B) [22], mediated by the enzyme M.HpyAVIII. We calculated that the other two cytosine MTases in N6 (T^m4^CTTC- or ^m5^CCTC-methylating) would only amount to about 0.8% methylation contribution each per all genomic cytosines. In the present study, we initially performed bulk detection of methylated cytosines of bacterial DNA of different *H. pylori* strains and mutants, shown for N6 wild type and two isogenic MTase mutants (Fig. 1C), using an ELISA-like setup with an ^m5^C-specific antibody. Using this method, we reproducibly detected quantitative differences of overall methylation in bulk (Fig. 1C), which was ^m5^C-specific and lacking in the GCGC mutant, although the detection was at low sensitivity, and not at motif-specific resolution. ^m4^C-specific methylation was not detected by this method (Fig. 1C).

### Global and single-base local quantitative resolution of genome-wide cytosine methylation detection at G^m5^CGC motifs using EM-Seq in *H. pylori*

**Fig. 2:**
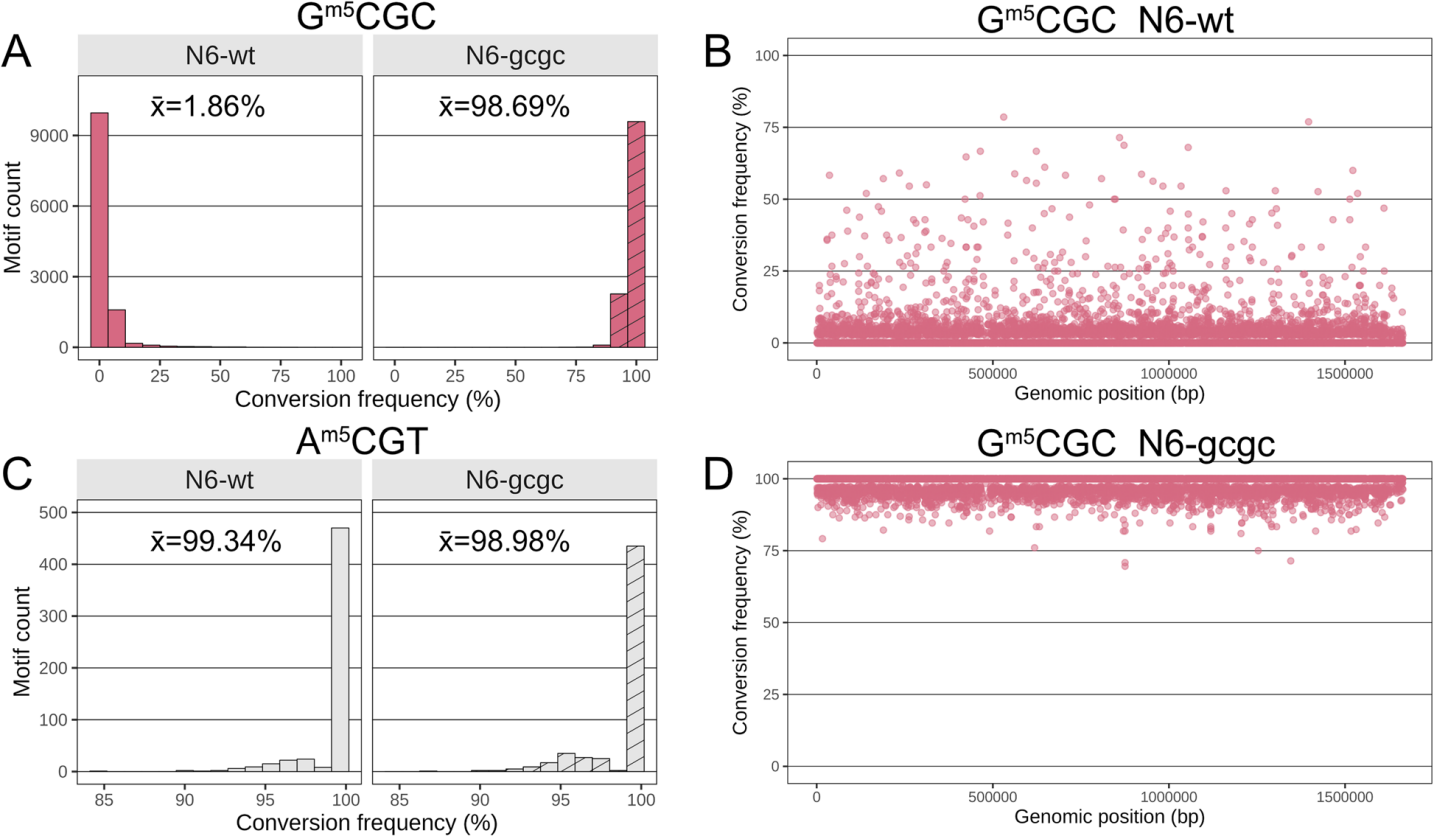
Genome-wide single-base resolution quantitative detection of methylated cytosine motifs in the *H. pylori* genome using EM-Seq. Comparing DNA methylation detected by EM-Seq methodology between *H. pylori* N6 wild type (N6-wt) and its isogenic GCGC MTase (M.HpyAVIII) mutant. Cytosines detected as converted are non-methylated, and non-converted cytosines are methylated. Higher conversion frequencies (calculated as average conversion frequency per motif count, summarized for all genomic motifs) indicate lower cytosine methylation. Experiments were performed in two replicates each, R1 and R2 (Table 1, Additional file 3: Table S2). **A** Bar graph of conversion frequency [%] per single motif for the GCGC motifs (motif counts on *y*-axis) in N6 wild type (N6-wt [replicate R1]) versus its isogenic HP1121 (M.HpyAVIII MTase) insertion mutant strain (N6-gcgc [replicate R1]), cultured on plates. **B** Dot plot of single-nucleotide resolution conversion frequency for each cytosine in GCGC motifs detected along the entire genome for wild type strain N6, same sample as in A, replicate R1. Conversion frequency (average [%]) for each motif cytosine is marked as one dot along the length of the whole genome (*x*-axis, labelled for genomic position). Low conversion frequency marks high methylation of the respective cytosine. Seventy-five percent of all cytosines in GCGC motifs were found to be 0% converted (quantitatively at 100% methylated). See also Fig. 7 and Additional file 3: Table S2 (single-base data) for more detailed analyses. **C** Bar graph of genome-wide conversion frequency for the ACGT motifs (which are not methylated in the N6 strain, due to absence of a motif-specific active MTase) in N6 wild type [R1] and its isogenic HP1121 mutant strain (N6-gcgc), same as in **A**, replicate R1. Motifs in bars were assigned according to their conversion frequency. **D** Dot plot of single-nucleotide conversion frequency for each cytosine in GCGC motifs detection along the entire genome for the *H. pylori* N6 M.HpyAVIII mutant (N6-gcgc [replicate R1]) cultured on plates. Conversion frequency (average [%]) for each motif cytosine is marked as one dot along the length of the whole genome (*x*-axis, labelled for genomic position). Low conversion frequency marks high methylation of the respective cytosine. We achieved > 95% average conversion detection for all relevant motif cytosines in this mutant, as one quality parameter of the method. In panels **A** and **C**, all single-motif-located cytosines of similar conversion frequency [%] were pooled into one bar (bins of 15); average percent conversion (*x*) of all respective motifs are indicated at the top of the graphs. Dot plot graphs at single-base resolution for the three methylated cytosine motifs along the genome in wild type (26695 strain) are included in Additional file 1: supplemental Figure S1

**Fig. 3:**
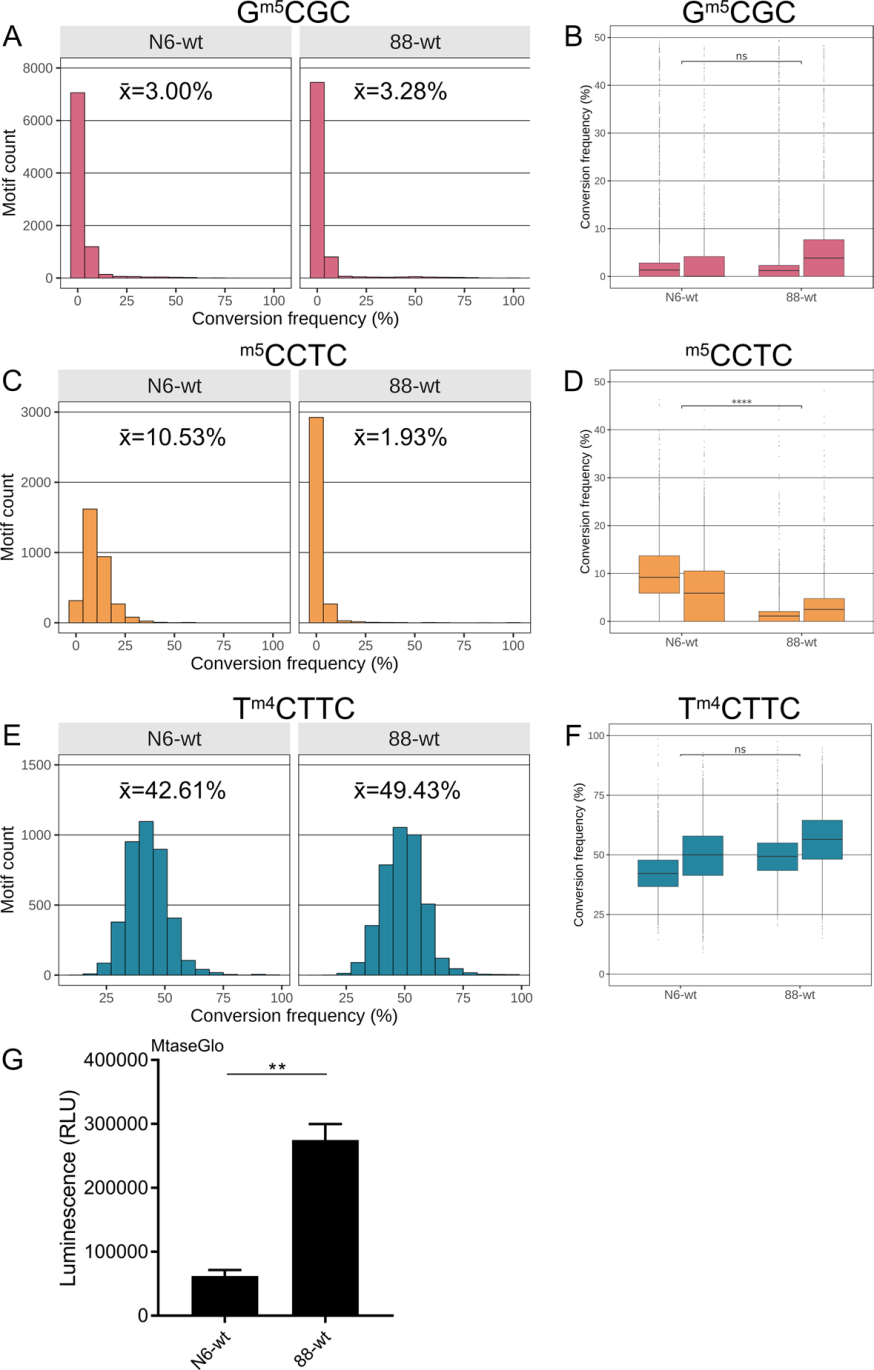
Comparing global averaged genome-wide DNA methylation at single-site resolution by EM-Seq between two different *H. pylori* wild type strains. **A, C,** and **E** show bar graphs for conversion frequency per genomic motif (EM-Seq experiments were performed in two biological and technical replicates [R1, R2]; one experiment for each strain, *H. pylori* N6 (N6-wt [R2]) or *H. pylori* 26695 (88-wt [R2]) shown); **B, D, F** show box plots with whiskers and statistics summarized for two biological and technical replicates (R1 and R2, see also Table 1 and Additional file 3: Table S2 for full conversion data) of each wild type strain (grown on plates). **A** and **B** show genome-wide results for methylation of the GCGC motifs (shown as conversion frequency [%]), **C** and **D** for the CCTC motif, and **E** and **F** for the TCTTC motifs. Chi-square *p* values for statistical significant differences between strains: * < 0.05 ** < 0.01 *** < 0.001 **** < 0.0001. Methylation of the two wild type strains was significantly different for the genome-wide quantitative single-nucleotide methylation average of CCTC motif methylation. In panels **A**, **C**, **E**, average percent conversion frequencies (*x*) for each experiment are highlighted at the top of the respective graphs. See Fig. 1B and Additional file 1: Fig. S1 for single-nucleotide resolution analysis of methylation for the three methylated cytosine motifs along the genome in the 26695 wild type strain (dot plots). Plots A, B, C, D, E, F are based on the conversion frequencies of all motifs shared between N6 and 26695 (88-wt), mapped to the 26695 genome (“ Methods”). Panel **G** depicts results of an MTase-Glo assay, performed in technical triplicates, showing significant differences in the SAH content (proxy of MTase activity) of the two different *H. pylori* wild type strains. Student's t-test ** *p* < 0.01

**Fig. 4:**
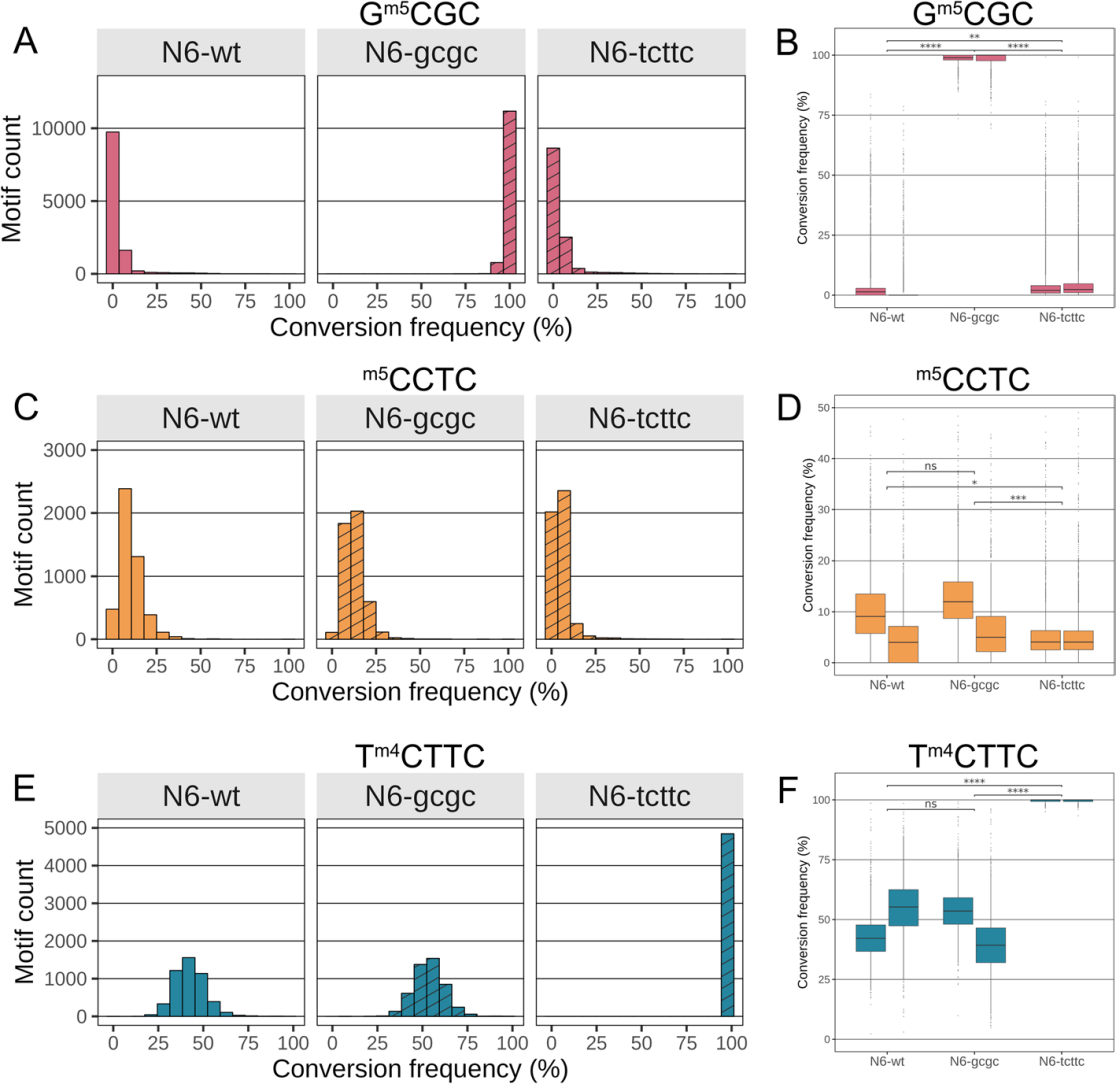
Comparing global averaged DNA methylation by EM-seq methodology in three different motifs for MTase mutants demonstrates significant differences for the single, methylated motifs between wild type and isogenic MTase mutants. **A** Bar graph comparison of genome-wide conversion frequencies for the GCGC motif in N6 wild type strain (N6-wt [replicate R2]) and both isogenic MTase mutant strains (N6-gcgc [R2]; N6-tcttc [R1]). **B** Statistical differences for the aggregated genome-wide single-base quantitative results of GCGC motif methylation between strains (two biological and technical replicates [R1, R2] for each strain were performed and are summarized). **C** Bar graph comparison of genome-wide conversion frequencies for the CCTC motif in N6 wild type strain and both MTase mutants as in A. **D** Statistical differences for the aggregated genome-wide single-base results of CCTC motif methylation between strains (two biological and technical replicates each). **E** Bar graph comparison for the genome-wide conversion frequencies for the TCTTC motif in N6 wild type strain and both MTase mutants, replicates as in **A**. **F** Statistical differences for the aggregated genome-wide single-base quantitative results of TCTTC motif methylation between strains (two biological and technical replicates each, see also Table 1 for replicate numbers; Additional File 3: Table S2 for full single-base conversion data). Chi-square *p*-value * < 0.05, ** < 0.01, *** < 0.001, **** < 0.0001. n.s. is non significant

In order to obtain more comprehensive and higher-resolution genomic information about cytosine (^m5^C, ^m4^C) DNA methylation in *H. pylori* [22], we then turned to a recently established methodology for human DNA, NEBNext Enzymatic Methyl Sequencing (EM-Seq). EM-Seq involves a direct DNA methylation detection at cytosines on primary, isolated DNA, which is aided by a two-step enzymatic protection of methylated cytosines and the subsequent enzymatic deamination and conversion of non-methylated cytosines to thymidine (“ Methods” and schematic in Additional file 1: Fig. S1A; [29]). The initial questions we asked in this study were how reliably and quantitatively the EM-Seq method can detect ^m5^C and ^m4^C modifications in bacterial DNA, using *H. pylori* (strain N6, Additional file 2: Table S1) as model organism. As a negative control strain, we used a newly constructed isogenic GCGC MTase insertion-inactivation mutant of strain N6, in which the G^m5^CGC-specific MTase (M.HpyAVIII; encoded by the HP1121 gene in strain 26695) has been inactivated. Upon EM-Seq treatment of purified DNA, converted library preparation, whole-genome sequencing and mapping (“Methods;” Additional file 1: Fig. S1A, S1B; Additional file 2: Table S1), we were able to analyze the methylation status (Additional file 1: Fig. S1C, Additional file 3: Table S2) of genomic cytosines at high resolution, for most samples detecting 99.9% of all cytosines (Table 1), including those located in known and predicted MTase target motifs [13]. We successfully established adequate quality criteria for the technique (“Methods,” and Table 1), partially based on comparison of the N6 wild type with its isogenic GCGC MTase mutant and spike-in controls, such as the detection of > 99% total cytosine calls, more than 96% methylation rate of methylated positive control DNA (pUC19 plasmid) and less than 3% methylation (more than 97% conversion) of non-methylated lambda DNA. Converging with previous experience with eukaryotic DNA [29], at least a 20-fold genome coverage was required for reliable quantitative detection of single-motif methylation (Methods). This setup yielded average conversion frequencies of 2–3% (corresponding to an average single-base quantitative methylation of 97–98%) for the GCGC motifs in the N6 strain genome (Fig. 2A, and Fig. 3A for a second biological replicate), while the average conversion frequency for the same motif was 98.7% in the N6 GCGC MTase-deficient mutant (Fig. 2A, Table 1, Additional File 3: Table S2). We achieved single-nucleotide resolution quantitation (average percent of conversion per each motif) of methylation for the GCGC motifs in the wild type strain, as depicted as a dot plot in Fig. 2B and as average per-site conversion values in Additional file 3: Table S2. All single GCGC motif cytosines were consistently methylated (≤ 95% converted, in both biological replicates; Additional file 3: Table S2), at variable single-base average values (local methylation frequencies). Other, predicted non-methylated, cytosines (i.e., those not part of GCGC, TCTTC and CCTC motifs), for instance in the A^m5^CGT motif, which is predicted not to be methylated in strain N6 due to a lack of the respective enzyme activity (Fig. 2C), were reproducibly > 97% converted on a single-base level in both wild type and GCGC mutant bacteria DNA and thus confirmed as non-methylated, emphasizing the high accuracy of the method (Fig. 2C and Table 1). In the GCGC (MTase M.HpyAVIII) mutant strain, we reproducibly did not detect the GCGC motifs as methylated (98.7% average single-base conversion; Fig. 2D, Additional file 3: Table S2; statistics for global methylation comparison of two biological experiments with sequencing in Fig. 4B).

**Table 1:** List of EM-Seq experiments and quality control (QC) parameters

*H. pylori* strain/mutant ^a^	Mean coverage (mean read depth/site)	Total cytosines coverage (%)	pUC19 conversion frequency (%) (methylated control)	Lambda Conversion frequency (%) (non-methylated control)	Culture media
88-wt-R1	92	97.38	1.6	99.1	Plate
88-wt-R2	> 100	97.58	1.1	99.5	Plate
N6-wt-R1 (N6-wt1)	> 100	99.97	0.4	99.5	Plate
N6-wt-R2 (N6-wt2)	> 100	99.98	1.5	99.4	Plate
N6-gcgc-R1	> 100	99.97	0.8	98.9	Plate
N6-gcgc-R2	98	99.95	1.3	99	Plate
N6-tcttc-R1	> 100	99.98	1.8	99.4	Plate
N6-tcttc-R2	> 100	99.98	1.7	99.2	Plate
N6-wt-OD0.5-R1	> 100	99.97	2.7	99.1	Liquid BHI standard
N6-wt-OD1.0-R1	77	99.96	1.1	98.5	Liquid BHI standard
N6-wt-OD0.5-R2	> 100	99.97	2.3	98.7	Liquid BHI standard
N6-wt-OD1.0-R2	> 100	99.98	3	99	Liquid BHI standard
N6-luxS-cl7-4h^b^-R1	> 100	99.98	1.6	99.2	Liquid BHI standard
N6-luxS-cl7-4h^b^-R2	> 100	99.96	1.8	98.4	Liquid BHI standard
N6-wt-4h^b^-R1 (N6-wt3)	> 100	99.97	1.3	99.4	Liquid BHI standard
N6-wt-4h^b^-R2 (N6-wt4)	> 100	99.98	1.4	99.4	Liquid BHI standard
N6-wt + Met-4h^b^-R1	44	99.93	1.4	99.3	Liquid-methionine-defined
N6-wt + Met-4h^b^-R2	> 100	99.98	1.4	99.6	Liquid-methionine-defined
N6-wt-WOmet-4h^b^-R1	> 100	99.97	2.4	99.1	Liquid-methionine depleted
N6-wt-WOmet-4h^b^-R2	> 100	99.98	2.8	98.8	Liquid-methionine-depleted

^a^Used strains are N6 (NCBI PRJEA89447) and 26695 (alias 88) (NCBI PRJNA175543), *luxS:* isogenic strain N6 *luxS* gene insertion inactivation mutant (clone 7), *gcgc*: isogenic strain N6 insertion mutant in MTase gene M.HpyAVIII, *tcttc:* isogenic strain N6 insertion mutant in MTase gene M2.HpyAII; all strains and conditions were tested in two biological and technical replicates, R1 and R2

^b^Liquid culture with a start O.D._600_ of 0.8, further grown with shaking for 4 h

### EM-Seq comprehensively detects differential global and local quantitative methylation at single-base resolution at the three different methylated cytosine motifs, for ^m5^C and ^m4^C

In the N6 wild type strain, the two other predicted cytosine-methylated motifs in addition to GCGC, TCTTC ([^m4^C] methylated by M2.HpyAII), and CCTC ([^m5^C] methyl-modified by M.HpyAVI enzyme), were also reproducibly detected as methylated (differentially protected from conversion) (Fig. 3). When compared to the GCGC motif (Fig. 3A, B), CCTC motifs were on average also strongly quantitatively methylated, both at the genome-wide average and at single-base level (7.76% overall average conversion; Fig. 3C, D; Additional file 3: Table S2), but with slightly reduced methylation at a single-nucleotide quantitative level (Additional file 3: Table S2). As for each single CCTC motif, we found all except one to be consistently methylated in wild type bacteria (Additional file 3: Table S2). The single cytosine in a CCTC motif that was reproducibly identified as non-methylated (100% converted) was located in an upstream region of a gene of unknown function and homology (locus tag N6_01411, site 1,426,309; Additional file 2: Table S1). In contrast, the ^m4^C methylated motif TCTTC displayed lower methylation both at the global and the local quantitative average levels than the other two motifs (Additional file 3: Table S2). Overall, the conversion frequency of genome-wide TCTTC motifs was at a 48.76% quantitative average (Fig. 3E, F; Additional file 3: Table S2). For the single TCTTC motifs, also all motifs but one were reproducibly detected as methylated (Additional file 3: Table S2). One single cytosine at site 546,255 in a TCTTC motif, in a CDS (locus tag N6_00526; Additional file 2: Table S1), which encodes an LPS keto-desoxy-octonate hydrolase subunit [32], was consistently not methylated (> 95% converted in all biological replicates).

We next addressed the reproducibility of the local single-base quantitative methylation results between different biological replicates. Therefore, we performed a cluster analysis on all the data points of single-site quantitative methylation (Additional file 3: Table S2) for all three cytosine motifs (“ Methods”), including four biological replicates of wild type *H. pylori* N6 grown under standard growth conditions. The clustering yielded different clusters of (i) reproducibly high-methylated, (ii) less reproducibly and differentially methylated (average intermediate single-base methylation), and (iii) reproducibly low-methylated local and unique sites for all three different cytosine motifs (Additional file 3: Table S2, Additional file 1: Fig. S2). Notably, for each of the three motifs in the wild type, we consistently detected clusters of reproducibly low/hypo-methylated cytosines in the respective motifs, which we analyzed separately. They comprise genes with an average higher quantitative single-site cytosine conversion (lower methylation) of 49.8% (GCGC motif), 40.3% (CCTC motif), and 71.6% (TCTTC motif), which, for each cluster, is significantly higher (see “ Methods”) than the genome-wide conversion average (Additional file 3: Table S2). Genes belonging to those clusters are summarized in Additional file 4: Table S3. While we cannot fully interpret the results right now, due to the complex biological networks and functional implications, we note that conspicuous consistently hypo-methylated sites are located in a number of genes of the virulence-associated *cag* pathogenicity island [33], in particular for the TCTTC and the CCTC motif sites. Further functions of CDS containing consistently hypo-methylated sites comprises cell division functions, central transcription factors, central metabolism, DNA restriction/methylation, and motility, including motility regulators (Additional file 4: Table S3).

### Comparative genome-wide cytosine methylation in two different *H. pylori* strains at single-base resolution using EM-Seq

We next compared quantitative genome-wide information about cytosine methylation at single-base resolution for two different wild type strains, N6 [34] and 26695 (88-wt) [13, 35] for each individual methylation site of any specific motif in the genome, as was obtained by EM-Seq methodology. Due to its predicted set of active DNA MTases [22], strain 26695, like strain N6, is expected to methylate only three sequence motifs at cytosine positions, GCGC (^m5^C), TCTTC (^m4^C), and CCTC (^m5^C). The single-site average quantitative datasets obtained by EM-Seq analysis for the three different cytosine-methylated motifs in two biological replicates each differed substantially within each strain: while all three motifs were abundantly methylated across the genomes (Fig. 3, Additional file 1: Fig. S1B, S1C, S1D), GCGC was always the most densely methylated motif, with all relevant cytosines reproducibly more than 96% protected from conversion (Fig. 3A, B), at about 3% single-site average C-T conversion for strain N6 and 3.28% conversion for strain 26695, which was a non-significant difference over two biological replicates between strains. The CCTC motifs, at single-site resolution, were also strongly methylated, however with statistically significant inter-strain differences (all motifs were on average ca. 7% less methylated (+ 7% more converted) in strain N6) (Fig. 3C, D, Additional file 3: Table S2). The TCTTC MTase (M2.HpyAII) target motifs showed a distinctly different (lower) single-base quantitative motif methylation pattern in both strains, with a peak of detection at an average of 40 to 50% conversion for the motifs genome-wide (Fig. 3E, F), and without statistical between-strain difference. Comparing the transcript levels of the three MTases revealed the highest transcript amounts for M.HpyAVIII (gene HP1121) (Additional file 1: Fig. S3B), and about one log lower transcript levels for both M.HpyAVI (HP0051) and M2.HpyAII (HP1368) in strain N6 (Additional file 1: Fig. S3D). The absolute transcript amounts of the three MTases were overall lower in strain 26695 than in strain N6 under standard growth conditions (not shown). Intra-bacterial methyl donors for MTases are usually delivered to the respective biosynthesis pathways as S-adenosyl methionine (SAM, AdoMet), which is then cleaved and converted into S-adenosyl-homocysteine (SAH). Measuring SAH in cell lysates as a proxy for MTase activity and general SAM degradation, revealed much higher SAH content for strain 26695 (88-wt) (Fig. 3G), which strain showed significantly higher M.HpyAVI-dependent methylation than strain N6.

### Loss of methylation in *H. pylori* MTase mutants affects quantitative methylation frequencies at unrelated motifs

We next tested whether loss of methylation in one abundant motif might influence modification of the two other cytosine motifs. In the GCGC MTase mutant (deficient in HP1121; M.HpyAVIII), we detected and quantitated methylation at the other two MTase target motifs, TCTTC and CCTC, with high confidence at single-base quantitative resolution, similarly as in the wild type (Fig. 4, Additional file 3: Table S2). When comparing quantitative methylation at the two unrelated motifs CCTC or TCTTC between the N6 wild type strain and the isogenic GCGC MTase mutant (Fig. 4), no significant differences in genome-wide average quantitative methylation were revealed (Fig. 4A to F, Additional file 3: Table S2), although we had hypothesized that loss of methylation in one abundant motif might influence modification of the two other cytosine motifs. Therefore, in addition to the GCGC mutant, we then compared another unrelated cytosine MTase mutant. Due to its interesting pattern of genome-wide average hypo-methylation, we selected the T^m4^CTTC-specific ^m4^C MTase M2.HpyAII and generated an allelic exchange mutant in strain N6. As expected, in the isogenic HP1368 (M2.HpyAII) mutant, we detected no methylation (> 99% global average conversion) of cytosines in TCTTC motifs (Fig. 4E, F, Additional file 3: Table S2). Surprisingly, in this mutant, the average single-base resolution quantitated methylation of both the GCGC and the CCTC motifs was significantly changed (more average conversion, meaning less methylation, for the GCGC motifs, less average conversion, meaning more methylation, for the CCTC motifs), compared to the parental wild type (Fig. 4B, D). This result was also reproducibly obtained using two independent biological and technical replicates. (Fig. 4). We speculate that this might be due to differential regulation of the enzymes or enzyme activities in the mutant, highlighting the possibility of crosstalk between different methylation systems.

### EM-Seq detects growth-phase-dependent differential methylation levels at TCTTC and CCTC motifs in *H. pylori*

**Fig. 5:**
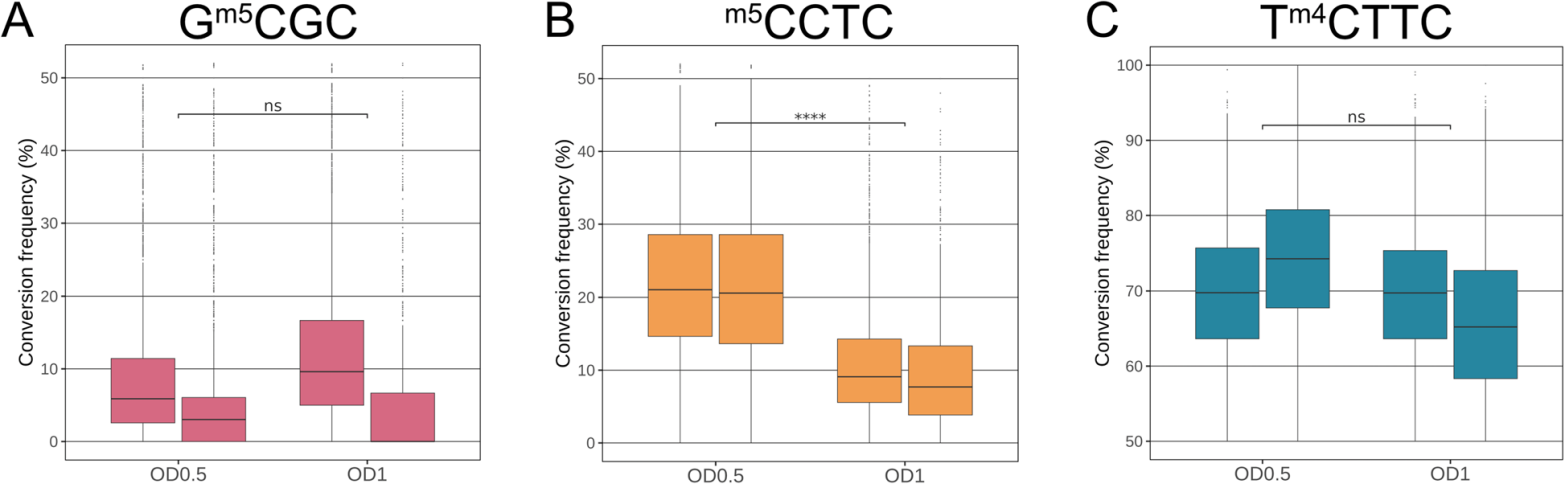
Growth phase-dependent averaged genome-wide cytosine methylation changes in *H. pylori* detected by EM-Seq*. H. pylori* strain N6 wild type (N6-wt) was cultured in liquid medium and harvested at two different points during the growth phase, O. D._600_ = 0.5 (early log phase) and O.D._600_ = 1.0. (mid-log phase). Two biological and technical replicates were analyzed for each condition (see Table 1). Box plots with whiskers are shown for each experiment containing replicate summaries [R1 and R2] of the averaged single-motif quantitative cytosine methylation from the EM-Seq experiments (shown as conversion frequencies [%]; data in Additional file 3: Table S2). **A** Motif GCGC analysis; **B** Motif CCTC analysis; **C** Motif TCTTC analysis. Chi-square *p*-values for significance of differences between growth phases: * < 0.05, ** < 0.01, *** < 0.001, **** < 0.0001

We further used the EM-Seq approach to address whether global and local quantitative methylation at the three cytosine motifs varied during different growth phases. We assayed three different growth phases in liquid culture, corresponding to OD_600_ = 0.5, (early log phase), OD_600_ = 1.0 (mid-log phase), and OD_600_ = 1.5 (mid- to late-log phase, data not shown). For the stringently methylated motif GCGC, the global quantitative differences averaged over all motifs and taking into account two biological replicates each, were not significant between growth phases (Fig. 5A). Our analysis using the same replicate samples yielded, however, different averaged quantitative results during exponential growth for the CCTC and TCTTC cytosine methylation motifs (Fig. 5B, C). We detected an increase of average single-site methylation (i.e., decrease of conversion %) between two harvesting time points, O.D._600_ of 0.5 and of 1.0, which was statistically significant for the CCTC motif (Fig. 5B). While we did not yet perform a comprehensive growth analysis nor a full analysis of the local quantitative methylation during growth, we noted genome-wide single-base quantitative changes in methylation frequency between the two harvesting time points for all motifs (Additional file 3: Table S2).

### EM-Seq analysis of *H. pylori* at different concentrations of the methyl donor, methionine, or in isogenic mutants deficient in methyl donor homeostasis reveals a differential influence on the methylome

*H. pylori* likely requires a high amount of methyl donors, converted into SAM, for their extensive genome methylation. We therefore hypothesized that acquiring a sufficient amount of methyl donors is an important housekeeping trait of the bacteria, affecting global and local methylation. SAM is thought to be provided to the bacteria through conversion of methylogenic amino acids (such as methionine), or, in some bacteria, regenerated using SAH recycling towards methionine via homocysteine, using MetH or MetE enzymes [36, 37]. *H. pylori* was reported to lack MetH and MetE enzymes [38–41], leaving amino acid/methionine uptake as the most likely and dominant source of methyl donors.

**Fig. 6:**
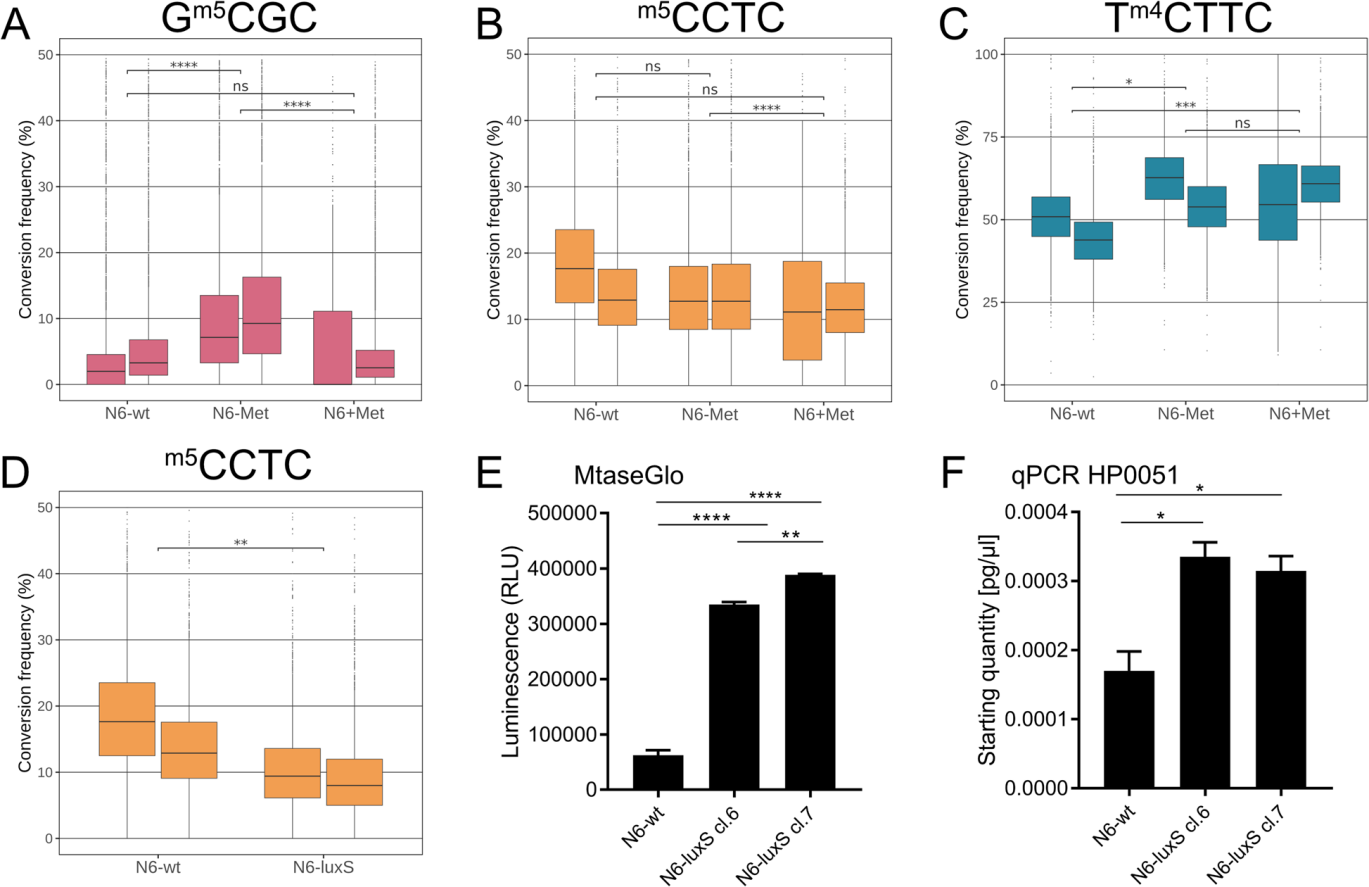
Quantitative DNA methylation of genome-wide cytosine motifs in *H. pylori* wild type compared for different culture conditions that impair methyl donor homeostasis.** A** to **C** show the comparison for the genome-wide aggregated quantitative data of the three cytosine-methylated motifs for *H. pylori* N6 under conditions of methionine (methyl donor) supplementation (methionine-replete) or methionine depletion. N6-wt designates standard liquid culture condition for the *H. pylori* N6 strain; N6-Met indicates methionine-depleted liquid medium; N6 + Met indicates methionine-depleted medium supplemented with a defined methionine concentration (50 µg/ml methionine). Box plots with whiskers were prepared summarizing the genome-wide cytosine methylation of each single genomic motif (as conversion frequency in [%]) for each condition (average for each site, summarized in box, with median). **A** GCGC motif; **B** CCTC motif; **C** TCTTC motif. Two biological and technical replicates are included for each condition. Chi-square *p*-values for significant differences between conditions: * < 0.05, ** < 0.01, *** < 0.001, **** < 0.0001. **D** Genome-wide averaged quantitative DNA methylation of CCTC cytosine motifs in *H. pylori* wild type (N6-wt) compared to an isogenic mutant in *luxS* (N6-luxS) in liquid medium. Box plot with whiskers for CCTC motif methylation (shown as conversion frequency [%]) is depicted including two biological and technical replicates of each strain. Chi-square *p*-values for significant differences between strains: * < 0.05, ** < 0.01, *** < 0.001, **** < 0.0001. **E** MTase-Glo assay for quantitating MTase activities in bulk between the N6 wild type strain (N6-wt) and the isogenic *luxS* insertion mutants (N6-luxS), shown for two independently obtained *luxS* mutant clones (cl.6 and cl.7). Background luminescence of a control condition without bacterial lysates was subtracted. **F** qRT-PCR for transcript quantification of MTase gene HP0051 (M.HpyAII), encoding MTase which methylates the CCTC motif), normalized against 16S rRNA transcript, in *H. pylori* N6 wild type and *luxS* mutant clones (two clones shown; three biological replicates were performed in technical triplicates [6 replicates] and summarized for each strain), which was increased in the mutants, corresponding to increased cytosine motif CCTC methylation shown in panel **D**. Full single-base conversion data in Additional file 3: Table S2

Using EM-Seq, we studied the genome-wide cytidine-motif methylations in *H. pylori* wild type, grown in liquid medium prepared with serum depleted for methionine (Methods), vs. in media supplemented with defined surplus concentrations of methionine (methionine-replete conditions, two biological replicates each). The data showed that methionine-replete conditions displayed a significant increase of quantitative genomic motif methylation for both GCGC and CCTC motifs when compared to methionine-low medium (Fig. 6A, B). The genome-wide average of single-base quantitative TCTTC methylation was not significantly different between methionine-depleted and defined replete conditions, while both conditions were significantly divergent to normal liquid medium (Fig. 6C). GCGC motif cytosine methylation had the most clear-cut association with methyl donor availability (methionine), as low methionine availability was significantly associated with genome-wide average lower cytosine methylation at those motifs (Fig. 6A). We also compared our standard liquid medium (BHI supplemented with yeast extract and 5% normal horse serum; “ Methods”) to the defined methionine-low- and methionine-replete liquid media. For the GCGC motif, methylation in standard liquid medium corresponded well to the results in methionine-replete medium, with higher overall methylation than the methionine-depleted condition, whereas for the other two cytosine motifs, the results were less comparable between standard (high-methionine) liquid medium and defined methionine-replete medium (Fig. 6).

In *H. pylori*, as in other bacteria, the enzyme LuxS is involved in the conversion of SAH to homocysteine, downstream of SAM cleavage. As the canonical methionine recycling pathway is not present in *H. pylori* [38–41], LuxS is reportedly still required for regenerating other amino acids from SAH and homocysteine, using a transsulphuration pathway [42], and possibly for the recycling of methionine via the methylthio-adenosine nucleosidase (MTAN) pathway [43]. Therefore, we also analyzed isogenic *luxS* mutants, which are predicted to not be able to perform crucial steps in downstream methyl donor metabolism, such as the conversion of SAH into homocysteine. We hypothesized that mutants deficient in LuxS could have effects on the intra-bacterial SAM-SAH homeostasis, for instance by accumulating SAH or S-ribosyl homocysteine (SRH), and by potential feedback inhibition of MTases. We generated isogenic allelic exchange mutants in the *luxS* gene of strain N6 and tested them using EM-Seq. The data showed that LuxS-deficient isogenic mutants displayed significantly increased global average and single-nucleotide-resolved local quantitative genome methylation at CCTC motifs in comparison to the wild type (Fig. 6D). We determined an increase in SAH for *luxS* mutants (two independent clones) over wild type using MTase-Glo assay (Fig. 6E). We also verified, using RT-qPCR, the transcript amounts of the HP0051 gene, encoding the CCTC MTase (M.HpyAVI), for wild type and *luxS* mutants. Here, we quantitated a strongly significant increase in specific transcript in the *luxS* mutants (four clones tested) in comparison to the wild type strain (Fig. 6F). At the other two motifs, GCGC, and TCTTC, our analyses did not reveal significantly altered cytosine methylation for the *luxS* mutants (Additional file 1: Fig. S3A and S3C), which matched the non-significantly different transcript amounts of the respective MTase genes (Additional file 1: Fig. S3B, S3D).

### Detailed analyses of genome-wide quantitative single-base localized methylation at cytosines by EM-Seq reveal differential methylation at overlap motifs

**Fig. 7:**
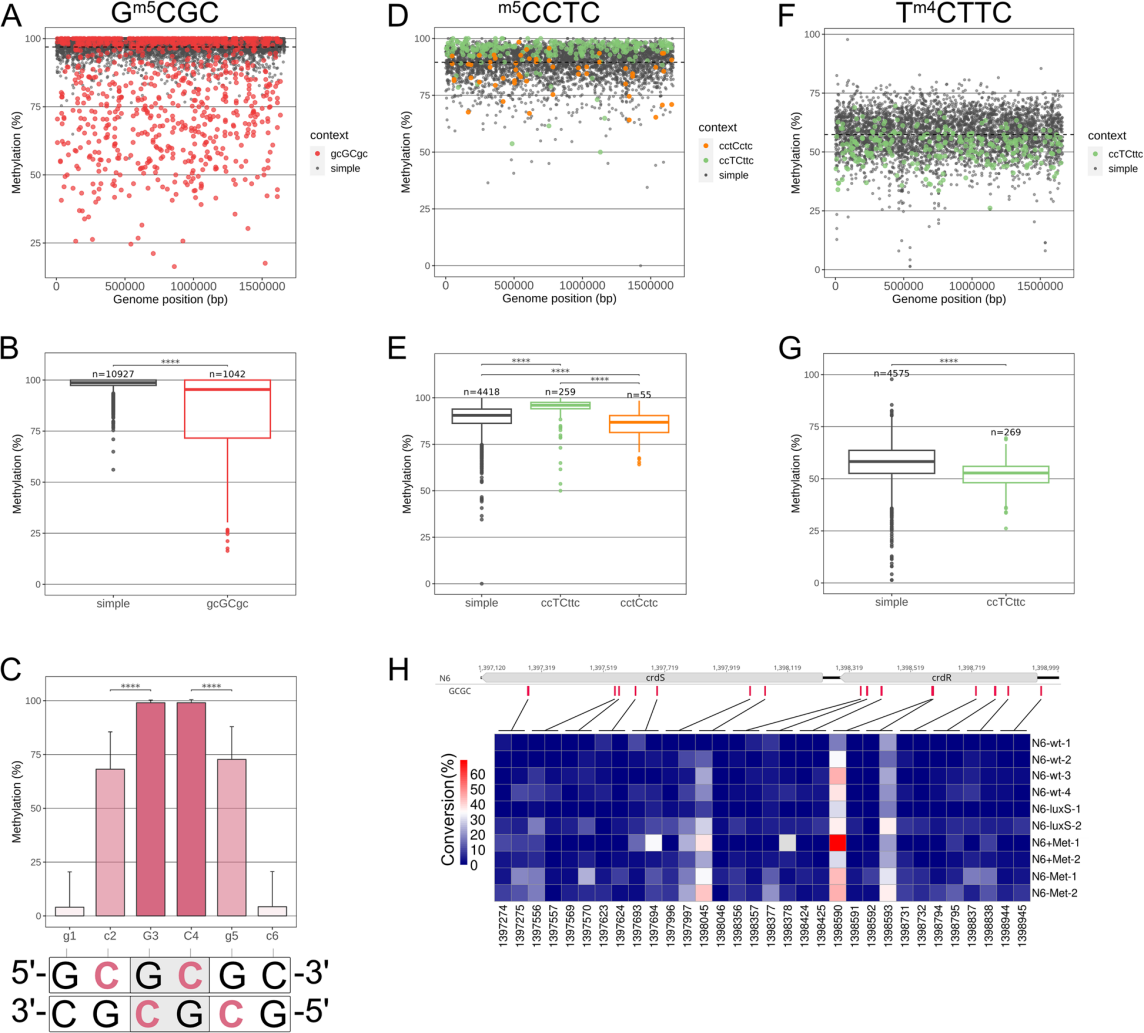
Genome-wide single-nucleotide resolution of cytosine methylation [%] in each of the three methylated motifs (GCGC, CCTC, TCTTC) in *H. pylori* N6. Dot plots (**A, D, F**) and box plots with whiskers (median) for the three different methylated motifs (**B, E, G**). Panels **A** (GCGC), **D** (CCTC), and **F** (TCTTC) show the genome-wide single-base resolution quantitative methylation for the three cytosine motifs; the nucleotide contexts for each motif, if occurring as simple motif or overlapping combined motifs, are marked as different dot colors. Panels **B**, **E**, and **G** show box plots comparing the combined individual site quantitative methylation levels between single GCGC, CCTC, and TCTTC motifs, and combined quantitative information on their overlapping motifs with other, homologous or heterologous, cytosine MTase target motifs. **A, B,** and **C** include the results for the average of methylation [%] in each single-motif cytosine for all genomic GCGC motifs, stratified according to single motif, or overlapping “doublet” GC**GC**GC motif. Bar graph in **C** indicates the average methylation, combined from genome-wide doublet motif analysis, for each methylated cytosine nucleotide on both strands of the palindromic doublet GC**GC**GC motifs. The dashed lines in **A**, **D**, and **F** indicate the mean methylation level for each motif. Statistical significance for datasets shown in **B**, **C**, **E**, and **G** was calculated using the Welch two-sample *t*-test (**** *p* < 0.001). For panels **A** through **G**, conversion data from N6 wild type replicate R3 (Table 1) were analyzed (data in Additional file 3: Table S2). **H** Heatmap matrix plot of methylation of all GCGC motif cytosines in gene cluster *crdR*/*crdS* (encodes a Two Component System for copper sensing and resistance). For each methylated base, data from five experimental conditions in two biological replicates are depicted (see Table 1; conversion data in Additional file 3: Table S2). The heatmap illustrates the robustness of lower methylation detection by EM-Seq for the flanking motifs of overlapping doublet GC**GC**GC motifs in comparison with single GCGC motifs within the genomic *crdS/crdR* gene cluster, whose transcript activity is regulated by GCGC methylation [16]. Methylation (shown as EM-Seq conversion [%] data) of each cytosine site is shaded in different colors, coded as in the graphic legend. While the 3’-upstream single GCGC motif in the promoter region of *crdR* is always high-methylated (low conversion, dark blue squares), cytosines in some overlapping GCGC motifs, in particular the flanking cytosines of a completely overlapping doublet within the CDS of *crdR*, exhibit reproducible lower methylation (high conversion, light blue, white and reddish squares; genomic positions 1,398,590 and 1,398,593; Additional file 2: Table S1). Single-nucleotide resolution quantitative cytosine methylation in GCGC motifs in panel **H** was compared over different standard and cytosine methylation-influencing conditions and mutants (luxS), including at least two biological and technical replicates for each condition (Additional file 3: Table S2)

Since, using EM-Seq, we were able to analyze quantitative differences in local methylation in the *H. pylori* genome at single-base resolution (Additional file 3: Table S2), we could now address more detailed questions pertaining to differences of individual single-base quantitative methylation levels at different cytosine-containing methylatable motifs for *H. pylori*. Analysis of the patterns for single-motif methylation in the wild type strain(s) so far revealed, for most specific target nucleotides and conditions, a low overall variation of genome-wide averaged quantitative methylation with the biological duplicates performed. However, we identified some particularly low-methylated sites, in particular for the GCGC or TCTTC motifs (Fig. 7, Additional file 1: Fig. S2, Additional file 4: Table S3). This raised the hypothesis that certain MTase target sites could be less accessible for MTase activity, which could be the case due to competitive effects, for instance with other MTases, or due to other bound proteins. Therefore, we next analyzed overlapping methylation target motifs, either of one single-motif type (e.g., GCGCGC), or of two combined motifs (e.g., CCTCTTC; Fig. 7) for their quantitative single-base methylation signatures. The location of such single and overlapping/combined motifs with methylatable cytosines in all three motifs along the *H. pylori* N6 genome is depicted in Additional file 1: Fig. S4. Differently located cytosines within the overlap sequences were significantly differentially methylated for all overlapping MTase motif combinations (colored dots in Fig. 7A, D, F). For two overlapping homologous GCGC or CCTC motifs, the central motif/cytosine was significantly more methylated than the two externally located motifs (Fig. 7B, C, E). For overlapping heterologous CCTC and TCTTC motifs (Fig. 7E, G), the innermost cytosine belonging to the TCTTC motif was significantly less methylated than a single TCTTC motif, while cytosines in the heterologous overlapping CCTC motifs were significantly more methylated than single CCTC cytosines (Fig. 7E). This finding may suggest interference by steric influences or competition between the latter two different MTases at two overlapping motifs. Comparison of the EM-Seq data with genome-wide ^m5^C methylation analyses using our own ONT data on N6 wild type confirmed a comparable pattern of average lower methylation at external cytosines of overlapping GCGC motifs (Additional file 1: Fig. S5), at ca. 90% correlation (Pearson test). In order to verify differentially methylated overlap motifs in specific genomic locations of known function, we then also checked known GCGC motifs in the promoter region and CDS of the *crdS/crdR* operon involved in copper resistance, which had been earlier reported to be transcriptionally regulated by the GCGC MTase M.HpyAVIII [16]. We found that all single methylated GCGC sites, including one previously identified site in the upstream promoter region of *crdR* (copper-sensitive response regulator of *crd* TCS; [16]), were reproducibly highly methylated (Fig. 7H). However, two overlapping GC**GC**GC motifs (sites 1,398,590 and 1,398,593; Additional file 2: Table S1) in the same genomic region, located within the open reading frame of *crdR*, were consistently, over several experimental conditions, found to be comparatively undermethylated (Fig. 7H). This distribution of low or high methylation average per single motif at those specific overlap sites was also robust and preserved between four replicates of *H. pylori* (N6) wild type in standard growth conditions, in low methionine media, or in a *luxS* mutant (Fig. 7H; Fig. S5, Additional file 1: Fig. S6). Consistently hypo-methylated GCGC cytosine sites, however, were not detectable at motifs within the upstream/promoter region of the *crd* operon (i.e., sites 1,398,944, 1,398,945, which were consistently highly methylated). We analyzed two additional genes of known function, which, like the *crd* operon, we had found before to contain GCGC motifs and to be differentially regulated in GCGC/M.HpyAVIII mutants [16]. Those two genes, *cah* (encoding alpha-carbonic anhydrase; [44, 45]) and *feoB* (encoding an iron uptake protein; [46, 47]) also contained consistently hypo-methylated GCGC overlap motifs, exclusively in their coding regions (Additional file 1: Fig. S6, Additional file 4: Table S3).

### EM-Seq identifies differentially methylated single-site cytosines in *H. pylori* under conditions of different methionine availability

It was obvious in our analyses that, in particular for the CCTC motifs, some of the consistently lower-methylated genomic cytosine sites (Additional file 4: Table S3) appeared not to be located in motif overlap regions (Fig. 7B, Additional file 4: Table S3). Therefore, we asked the question, whether we can also find reproducible and significant differential methylation at single-base resolution in some motif cytosines under certain defined culture conditions. We specifically selected the methionine-depleted and methionine-replete conditions (see Fig. 6), since we surmised that methionine availability is most directly involved in methionine homeostasis and possibly altered MTase activity. We performed statistical analysis by likelihood ratio chi-square test on the single-base quantitative EM-Seq data of all cytosines in coding regions in the three motifs (for all genomic sites, see Additional file 3: Table S2), between the methionine-depleted and methionine-replete conditions (two biological replicates each (Table 1)). Indeed, we found that a number of intragenic cytosines in all three motifs were significantly differentially methylated (with cut-off at a *p* value of ≤ 0.01) under those conditions, with some motif cytosines higher- and most motifs lower-methylated under low methionine conditions (Additional file 5: Table S4). Most of the differentially methylated cytosines (> 2000 sites) were indeed located in GCGC motifs, with more than one log_10_ less relevant extracted sites found for each of the two other motifs. The genes identified to harbor differential quantitative methylation at cytosines of all three MTase motifs belong to functionally different gene categories and located rather evenly over different parts of the genome. Notably, we were able to associate significant downmodulation of transcripts for *cah* and *feoB* to the methionine-depleted culture condition, using qPCR (Additional file 1: Fig. S6). This condition was also reproducibly associated with significantly differential methylation /hypo-methylation under conditions of methionine depletion (Additional file 5: Table S4) in some cytosine motifs (for instance, GCGC site 42,101 for *cah* and GCGC site 1,522,380 for *feoB*), within the coding regions of those two genes.

## Discussion

Various new methods aided by high-throughput sequencing have helped to deepen our understanding about epigenetic modifications in all kingdoms of life. For bacteria, this has recently led to discoveries of unsurpassed variation of epigenetic modifications and their possible functions and regulation [1, 13, 21, 22, 48–50].

While PacBio SMRT sequencing or, more recently, ONT sequencing have been used to broadly clarify the extent and motif specificity of methylation on nucleic acids, quantitative information on how intensively and how many of the target nucleotides of any type of MTase are modified at any given time point or condition (single-base-resolution quantitative information), in particular in bacteria, were largely lacking. Recently, bacterial genomes have been analyzed extensively by third-generation sequencing. This approach has generated new knowledge on quantitative and localized genome-wide nucleotide methylation, for instance during different phases of bacterial growth and development, in *E. coli* [51], *Klebsiella* [52], and *Caulobacter crescentus* [53]. Reproducibly hypo-methylated single bases have been identified in *Vibrio cholerae* and *Mycobacterium tuberculosis* genomes, which may be linked to phenotype variation in some pathogenic bacteria [54, 55]. Most studies on *H. pylori* so far have restricted their high-throughput sequencing-based epigenomic analyses to non-quantitative determination of genome-wide single-site methylation and MTase target motif discovery [13, 25]. These methods are still cost-intensive and all have limitations in detecting certain modifications. This has recently led to the development of other, enzyme-aided sequencing-based methods, in particular for ^m5^C modification, and to overcome the disadvantages of bisulfite sequencing. A recent study developed EM-Seq methodology to analyze single-nucleotide resolution quantitative information on CpG (^m5^C) methylation in human genomes [29] and further improved the method as SEM-Seq [56]. These developments prompted us to use the methodology to quantitatively analyze genome-wide and single-base quantitative methylation at cytosines in *H pylori*. Before, it was rather unknown whether bacterial genomes are rather uniformly methylated at single bases, and which protected areas or reproducibly hypo-methylated single nucleotides in bacterial genomes exist, although bacterial genomes are less packed and structured in comparison to eukaryotic, nucleosomally condensed, genomes. Moreover, the roles of environmental conditions and growth phase in governing genome-wide methylation patterns during the bacterial cell cycle have only started to be unravelled [51].

We have used EM-Seq methodology and addressed some open questions in the highly methylation-prone bacterium *H. pylori*, adapting the method for single-base quantitative resolution of methylation to bacterial whole genomes. Before, high-throughput Rapid Identification of Methylase Specificity (RIMS)-Seq sequencing, which is similar but not identical to EM-Seq, was employed to obtain some single-base-resolution quantitative information on human and bacterial genomes, including *H. pylori* [57]; however, the latter method was not able to detect ^m4^C methylation. We succeeded in obtaining high-quality whole-genome cytosine methylomes from two different *H. pylori* strains. At least 20- to 30-fold average coverage of the genomes was required to gain meaningful single-base resolution quantitative information. We also defined other quality criteria (partially on the basis of comparisons between wild type and an isogenic GCGC non-methylated mutant), such as the detection of > 99% total GCGC motif calls, more than 96% methylation of methylated positive control DNA (pUC19 plasmid) and less than 3% methylation (more than 97% conversion) of non-methylated lambda DNA control. Other unknown methylated cytosine motifs were not detected in the two strains analyzed.

Concerning single-nucleotide quantitative information, for the first time we were able to detect by enzyme-aided methylation detection sequencing (EM-Seq) significant quantitative differences, including at single-nucleotide resolution, in whole-genome cytosine methylation at the different known active methylated motifs of two *H. pylori* strains (Additional file 3: Table S2). Interestingly, the method was able to reliably detect ^m5^C as well as ^m4^C methylation (e.g., T^m4^CTTC modification) in the *H. pylori* genomes. ^m4^C methylation is understudied, but has been associated with gene regulation in the pathogenic bacteria *H. pylori* [17] and *Leptospira interrogans* [58]. In terms of single-base quantitative differences, all GCGC motifs were consistently methylated, and quite strongly methylated (> 95% of all genomic motif GC cytosines at more than 75% methylation). The ^m5^CCTC motifs were also strongly methylated at a global and local scale, showing a clear-cut growth phase and strain dependency. In contrast, the T^m4^CTTC motif was on average less methylated at a single-base resolution than the other two target motifs by its cognate enzyme, similarly along the whole genome (about 49% average single-base methylation). For both TCTTC and CCTC motifs, only one single site per genome for each motif was reproducibly not methylated, upstream of one CDS in an LPS-related gene, and in a second, unrelated CDS of unknown function, respectively. For all three motifs, we detected, using cluster analysis, a number of motifs within coding regions that were reproducibly and consistently hypo-methylated under various conditions. The genomic distribution of those open reading frames was scattered and functional categories were diverse, ranging from central metabolism to virulence factors, and will need further study with respect to their regulation and functional interrelatedness. Loss of genome-wide methylation at one motif (CCTC) was impacting on the quantitative global and local methylation at other motifs. However, the mechanisms remain to be investigated and are anticipated to be multifactorial.

It is unknown how a sufficient amount of methyl donors can be provided for the abundant MTase activities in *H. pylori*. As the canonical methionine recycling pathway is not present in *H. pylori* [59], it is a plausible possibility that those are mostly sourced by the uptake of methylogenic amino acids, such as methionine. When we supplemented defined excess methionine to methionine-depleted growth media, which is one way to introduce surplus methyl donors into *H. pylori*, this led to an overall average hypermethylation of numerous cytosine motifs, in particular for the GCGC and CCTC (^m5^C-methylated) cytosine motifs, in comparison to the methionine-depleted condition. Increased GCGC motif cytosine methylation had the most clear-cut association with methyl donor availability (methionine), as low methionine availability was significantly associated with lower cytosine methylation at a large number of those motifs. Isogenic *H. pylori luxS* mutants also showed a reproducible cytosine motif hypermethylation, particularly at ^m5^CCTC motifs. In strong concordance with this result, we obtained significantly elevated transcripts for the cytosine MTase, HP0051 (CCTC MTase, M.HpyAVI). The significant increase in SAM breakdown product SAH that we determined using the MTase-Glo assay is suggestive of an increased MTase activity in *luxS* mutants. Part of the observed increase in intra-bacterial SAH may be directly due to the loss of LuxS which is responsible for converting potentially toxic SAH within the bacterium [42]. Considering previous publications that point out strain-specific phenotypic differences for *luxS* mutants (motility, growth) [38–40, 60], and also potential effects of AI-2 downstream of LuxS [41, 60], despite the lack of AI-2 receptor in *H. pylori*, the overall effects and mechanisms of LuxS and LuxS-deficiency in *H. pylori* will require further in-depth study. Hypotheses and experimental conditions concerning methyl donor provisioning in *H. pylori* will also have to be addressed in more detail.

As for local, single-site resolution quantitative methylation analyses, we have opened some more avenues for further study. We found that consistently in an *H. pylori* wild type, overlapping methylation motifs, either of homologous motif type (e.g., GCGCGC), or of two different/heterologous combined motifs (e.g., CCTCTTC), led to the effect that the overlapping motifs were significantly differentially methylated. This finding, in the second case example, also suggested a competitive aspect between two different cytosine MTases at two overlapping motifs, in which the CCTC ^m5^C MTase M.HpyAVI seemed to be more competitive in binding or more processive than the TCTTC-methylating ^m4^C MTase. We identified numerous GCGC dual overlap motifs as consistently hypo-methylated. Some of those are located within CDS which were found previously as transcriptionally regulated by loss of GCGC MTase [16]. This effect was, for instance, prominent for the coding regions of four genes, *crdS*-*crdR*, *cah*, and *feoB*, which all contained hypo-methylated overlap motifs exclusively in their open reading frames but not upstream. While all lower-methylated cytosines in GCGC motifs were indeed located in dual overlap motifs, some consistently low-methylation cytosines were detected for CCTC and TCTTC motifs, which did not fall into the category of motif overlaps. When we compared EM-Seq data at single-base resolution under the best-defined conditions of methionine depletion and supplementation, we identified a number of differentially methylated individual cytosines for the three motifs, most prominently for the GCGC motifs, which matched the global average analysis of significant quantitative variation at this motif under the same conditions. Differentially methylated cytosines under those conditions are again localized in numerous coding regions associated to various functions, but do not necessarily match with consistently low-methylated cytosines. Interestingly, differentially methylated cytosines as per methionine availability were also identified in the open reading frames of the GCGC-regulated genes *crdS*, *cah*, and *feoB*. For some of the CDS which share differential methylation, functional groups are visible, for instance including several CDS each for cell division, motility, and the *cag* pathogenicity island. More detailed future investigation will be needed to dissect interconnectivity or co-regulation between the different functional categories.

## Conclusions

Taken together, we have evaluated the potential of the EM-Seq technology for the study of detailed bacterial epigenetics and used this approach to reveal novel global and local quantitative information about genome-wide cytosine methylation in *H. pylori* under various conditions. In the future, more detailed and data-rich local single-nucleotide resolution analyses of cytosine methylation, also in the context of other, non-cytosine MTase motifs, followed up by mechanistic and functional analyses, will have to be performed to further assess the potential, robustness, and limitations of the localized quantitative analyses by EM-Seq. Furthermore, the establishment of the EM-Seq method for bacteria and for *H. pylori* genome-wide analyses at single-base resolution will now allow to measure and interpret the quantitative outcome of various interventions into the bacterial cytosine methylome.

## Methods

### Bacterial strains and culture conditions

*H. pylori* wild type strains N6 [34] and 26695 [35] were thawed from cryo-stocks and cultured for only few passages on blood agar plates (blood agar base II; Oxoid, Wesel, Germany) supplemented with 10% horse blood (Oxoid) and the following antibiotics, amphotericin B [4 mg/liter], polymyxin B [3.2 mg/liter], trimethoprim [5 mg/liter], and vancomycin [10 mg/liter] (all purchased from Sigma-Aldrich, USA). The *H. pylori* mutants were cultured on blood agar plates either containing kanamycin (20 μg/ml) or chloramphenicol (20 μg/ml) as selective antibiotics as required, in addition to the non-selective antibiotics. Bacteria were passaged every 20 to 22 h on fresh plates, prior to setting up an experiment, thereby keeping passage numbers low. For growth of *H. pylori* in liquid cultures, brain heart infusion (BHI, Becton–Dickinson) medium with yeast extract (2.5 g/liter), supplemented with 5% heat-inactivated normal horse serum (Thermo Fisher Scientific—Gibco) was used. Both, agar plates and liquid cultures, were incubated in micro-aerobic conditions by using air-tight jars (Oxoid) and Anaerocult® C gas-generating bags (Merck). The genome annotations, nucleotide positions, and locus tags relevant for the EM-Seq analysis, based on our previously published draft genome of *H. pylori* N6 (NCBI PRJEA89447), are listed in Additional file 2: Table S1. *H. pylori* 26695 closed and annotated genome (NCBI PRJNA175543) was used to map EM-Seq reads from this respective strain and serve as a basis for strain comparisons.

### Growth curves of *H. pylori*

For long-term growth culture, an overnight pre-culture of *H. pylori* N6 wild type was set up in BHI medium (Oxoid) with 2.5% yeast extract and 5% horse serum. This pre-culture was used to set up a day culture for time course of growth (growth curve), with a starting OD_600_ of ∼0.15. Bacterial pellets were harvested at OD_600_ of 0.5, 1.0, and 1.5. For all other short-term co-incubation conditions, a short-term liquid culture was set up with an initial OD_600_ of ∼0.8 (prepared from a pre-culture on plate), then further cultured with shaking for 4 h, before harvesting pellets for DNA (EM-Seq analyses) and RNA isolation.

In order to grow *H. pylori* N6 in methionine-depleted versus -replete conditions in liquid culture, bacteria were resuspended in BHI medium, supplemented with 5% dialyzed Fetal Bovine Serum One Shot™ (Thermo Fisher Scientific—Gibco), which is depleted in amino acids, in either the absence or the presence (supplementation) of 50 µg/ml L-methionine (Merck) with an initial OD_600_ of ∼0.8, and then further cultured for 4 h.

### Cloning and *H. pylori* mutant generation

#### HP1121 MTase mutant

Methyltransferase mutants were generated in strain *H. pylori* N6 by natural transformation-mediated allelic exchange and disruption of the coding region by *aphA-3* cassette insertion, conferring kanamycin resistance. The mutant in gene HP1121 (GCGC motif-targeting MTase, M.HpyAVIII) was generated as described in [16], with a small deletion in the middle of the gene, replaced by inserting a kanamycin resistance cassette (*aphA-3*). The insertion was integrated into the *H. pylori* N6 chromosome by homologous recombination and allelic exchange. Clones were selected on kanamycin-containing plates and tested by PCR with a suitable combination of primers and by sequencing of the PCR products.

#### HP1368 MTase mutant

HP1368 (TCTTC motif-specific MTase, M2.HpyAII) gene was PCR-amplified from *H. pylori* N6 genomic DNA using primers hp1368_RT1 and hp1368_rv (Table 2). The PCR product was restricted with BamHI/KpnI and cloned into plasmid pUC18. The resulting plasmid, pCJ2100, was propagated in *E. coli* DH5α, and transformants were selected on LB agar containing ampicillin. Further, pCJ2100 was linearized by PCR amplification with BglII flanking primers hp1368_fw2 and hp1368_rv2 (Table 2). BglII-digested PCR product and BamHI-digested *aphA-3* cassette were ligated resulting in the disruption of hp1368 by *aphA-3* cassette and a deletion of 94 bp. The resulting plasmid (pCJ2101) was transformed into *H. pylori* N6, and mutants were selected on blood agar plates containing kanamycin. Partial deletion of hp1368 was subsequently confirmed by PCR and partial sequencing of the chromosomal insertion site.

**Table 2:** Oligonucleotide primers used for cloning and sequencing

Primer name	Sequence (5′ → 3′)	Description
hp1368_RT1	AAAAGGATCCGAATATCAATAAAGTGTTTTATC	Cloning of HP1368 into pUC18 and forward primer for qPCR of HP1368
hp1368_RT2	TGTGCCATTTTTTGCGTAATC	Reverse primer for RT PCR of hp1368
hp1368_rv	AAAAGGTACCTCAAAATCAAACAAGTTT	Cloning of HP1368 into pUC18
hp1368_fw2	AAAAAGATCTCTATAGAATTTATCGGCGTG	Reverse amplification and insertion of BglII restriction site for aphA3-III cassette cloning
hp1368_rv2	AAAAAGATCTGTAGCTCCCAAACATCAATC	Reverse amplification and insertion of BglII restriction site for aphA3-III cassette cloning
luxS-BamHI-fw	AAAAGGATCCGTGGAAAGTTTGGTGGGGAT	Cloning of HP0105 (*luxS*) and flanking regions into pUC18
luxS-KpnI-rv	AAAAGGTACCCTATTTTGACTTCTTTTGCAGAT	Cloning of HP0105 (*luxS*) and flanking regions into pUC18
luxS-BglII-fw2	AAAAAGATCTTTAAACCATGACAATTACACGG	Reverse amplification and insertion of BglII restriction site for aphA3-III cassette cloning
luxS-BglII-rv2	AAAAAGATCTGTGTAGGCTAGGCATGTCCA	Reverse amplification and insertion of BglII restriction site for aphA3-III cassette cloning
hp0051_RT1	GATTTATTTTGTGGGGCTGG	Forward primer for qPCR of HP0051 (CCTC MTase) gene
hp0051_RT2	GCGTTTTTATGGTTGTTTTCAAA	Reverse primer for qPCR of HP0051 (CCTC MTase) gene
hp1121_RT1	ATTGGTGGAGGCCGTTTGG	Forward primer for qPCR of HP1121 (GCGC MTase) gene
hp1121_RT2	TTGATTCGCATCAAATCCCCA	reverse primer for qPCR of HP1121 (GCGC MTase) gene
luxS-RT1	TGGATCACACCAAAGTCAAAG	Forward primer for qPCR of HP0105 (*luxS*) gene
luxS-RT2	TAGGCTAGGCATGTCCATGT	Reverse primer for qPCR of HP0105 (*luxS*) gene
feoB-RT1	GAGCGTAATTTAGCCTTAAGC	Forward primer for qPCR of HP0687 (*feoB*) gene
feoB-RT2	TTGGCACGCACACAACCCC	Reverse primer for qPCR of HP0687 (*feoB*) gene
cah-RT1	GCTGGGACAAATTGCACAAAG	Forward primer for qPCR of HP1186 (*cah*) gene
cah-RT2	GTGAAAAAGACCGCTTTAGGTT	Reverse primer for qPCR of HP1186 (*cah*) gene

#### *luxS* mutant

To inactivate *luxS*, we used a similar strategy as for HP1368 (see above section), *luxS* insertion inactivation mutagenesis was designed by first amplifying the *luxS* gene from *H. pylori* N6 genomic DNA, using BamHI and KpnI flanking primers luxS-BamHI-F and luxS-KpnI-R (Table 2). The PCR product was then BamHI/KpnI-digested and ligated into plasmid pUC18. The resulting plasmid, pCJ2113, was propagated in *E. coli* DH5α under ampicillin selection. For deletion of 100 bp and insertion of the *aphA3* cassette within the cloned *luxS* gene, pCJ2113 was linearized by PCR amplification with BglII flanking primers luxS-BglII-fw2 and LuxS-BglII-rv2. The PCR product was subsequently BglII-digested and ligated with BamHI-cut *aphA3’*-III (kanamycin) cassette. The resulting plasmid (pCJ2115) was transformed into *H. pylori* N6, and mutant clones were selected on blood agar plates containing kanamycin. Deletion of *luxS* was confirmed by PCR and partial sequencing. Growth curves of *luxS* mutant clones in strain N6 were performed in standard liquid media and mutants had slightly delayed growth in liquid in comparison to the wild type (own unpublished results). Therefore, direct comparisons of wild type and *luxS* mutants for genome-wide methylation were performed with cultures from a short-term (4 h) incubation in liquid broth (inoculated directly from freshly 1-day grown plates) at an OD_600_ of ∼0.8, in order to minimize the effect of growth impairment in liquid. All primers used for cloning and sequencing are listed in Table 2.

### RNA isolation

*H. pylori* RNA extraction was performed from 2 ml of bacterial cells grown in liquid medium to/at an OD_600_ of 0.8 (or as indicated otherwise in the figure legends). Bacteria were pelleted by centrifugation at 6000 × *g*, 3 min, 4 °C and immediately snap-frozen in liquid nitrogen to prevent RNA degradation. Pellets were then stored at − 80 °C. Bacterial pellets were disrupted in a Fastprep bead-beater (MP Biomedicals Inc., USA) at 6.5 m/s for 45 s using Lysing Matrix B 2-ml tubes containing 0.1-mm silica beads (MP Biomedicals, Eschwege, Germany). RNeasy Mini kit (Qiagen, Hilden, Germany) was used according to the manufacturer’s instructions for RNA isolation, following which the isolated RNA was DNaseI-treated using TURBO DNA-free™ Kit (Ambion, Kaufungen, Germany). Isolated and purified RNA was checked for the absence of DNA contamination by amplification of the 16S rRNA gene by PCR. RNA concentrations were determined using a NanoDrop 2000 spectrophotometer (Peqlab Biotechnologies), and RNA quality was assessed in an Agilent 4200 Tape Station system using high-sensitivity RNA Screen Tapes (Agilent, Waldbronn, Germany).

### RT-qPCR

For quantitation of gene expression by qPCR, cDNA was synthesized from 1 µg of DNAse-treated RNA using the SuperScript III Reverse Transcriptase (Thermo Fisher Scientific, Darmstadt, Germany) as described before, with suitable quality controls [16]. qPCR was performed in a BioRad CFX96 system with 4 nM of gene-specific primers (Table 2) and SYBR Green Master Mix (Qiagen, Hilden, Germany). The following cycling conditions were used: 95° 10:00, (95° 0:30, 55° 0:30, 72° 0:30, 40 cycles), melt curve 60° to 95°, increment 0.5° for 0:05. Reactions were set up in technical triplicates, including specific quantity standards for each target gene. Results were equalized to 1 µL of cDNA, and 16S RNA transcript amounts in the same samples were used for normalization across samples.

### MTase-Glo™ methyltransferase activity assay

The activity of MTases in vitro can be quantitatively determined by the conversion of SAM to SAH in vitro, in a reaction containing specific purified enzymes and using a bioluminescence-based assay, MTase-Glo™ Methyltransferase Assay (Promega, USA). We adopted the same method to measure the global intra-bacterial SAH content as a proxy for relative enzyme/MTase activity in a live bacterial sample. For this purpose, bacterial whole-cell lysates were prepared from *H. pylori* wild type and respective mutants, harvested from blood agar plates after 20 h of fresh growth. Bacteria were resuspended in 100 mM HEPES buffer solution (Thermo Fisher Scientific -Gibco) and lysed by sonication. The total protein content was first determined by BCA assay (Pierce, Thermo Fisher Scientific, USA), and absorbance was measured in a microtiter plate reader (Victor Nivo Multimode Microplate Reader, PerkinElmer). Two micrograms of total protein per each sample lysate was used for subsequent MTase-Glo™ methyltransferase Assay. Background values (baseline) of a reaction sample without the addition of lysate were subtracted from each reaction. Relative comparisons between strains and/or conditions were performed.

### Genome-wide DNA methylation characterization in bulk by m^5^C ELISA

DNA methylation ELISA was performed to detect genome-wide 5-methylcytosine (m^5^C) methylation. For this purpose, *H. pylori* genomic DNA was isolated using the DNeasy® UltraClean® Microbial Kit (Qiagen, Hilden, Germany) according to the manufacturer’s instructions. For bulk detection of methylated cytosines, briefly, 50 ng of *H. pylori* DNA in 50 µl of D-PBS was denatured by heating to 98 °C for 10 min and immediately placing the sample on ice for 10 min. The DNA was then added to a DNA-binding 96-well microtiter plate (# 467,320 Nunc, Roskilde, Denmark) and incubated at room temperature for 2 h. Following incubation, DNA was UV cross-linked to the plate for 30 s in a UV-crosslinker (Carl-Roth, Karlsruhe, Germany). The wells were then washed once with 250 µl washing buffer (1 × TBS containing 0.05% Tween 20) and blocked in 200 µl of blocking buffer (1 × D-PBS + 10% FCS) for 1 h. Wells were again washed three times and incubated with 100 µl of 1:350-diluted anti-5-methylcytosine mouse mAB (MABE146, Sigma-Aldrich) in blocking buffer for 1.5 h. Subsequently, the wells were aspirated and washed again three times. Following thereafter, the plate was incubated with 100 µl of 1:5000 dilution of horseradish peroxidase (HRP)-conjugated goat-anti-mouse polyclonal antibody in blocking buffer for 1 h. After five additional washes, 100 µl substrate reagent per well (TMB Substrate Reagent Set, BD Biosciences) was incubated for 30 min and the reaction was stopped by 50 µl 1 M H_2_SO_4_ (Sigma-Aldrich). Each DNA methylation ELISA was performed in triplicates for each sample. The final absorption was measured at 450 nm in a microtiter plate reader (Victor Nivo Multimode Microplate Reader, PerkinElmer) and the background absorption of the plate measured at 540 nm. Ultimately, due to a change of commercial antibody performance, this semi-quantitative detection method was no longer available.

### Enzyme-based genome-wide DNA methylation analysis by sequencing (EM-Seq)

Genome-wide, single-base resolution detection of DNA methylation at cytosine residues (m^5^C and m^4^C type) was achieved using the NEBNext® Enzymatic Methyl-Seq Kit (NEB #E7120S), in conjunction with the Illumina pyro-sequencing platform (MiSeq). The EM-Seq libraries were prepared each using 200 ng of *H. pylori* genomic DNA, isolated using the DNeasy® UltraClean® Microbial Kit (Qiagen, Hilden, Germany). All single libraries were tagged with individual barcodes. All steps were performed according to the manufacturer’s instructions with a few exceptions, as follows. Incubation time of the EM-Seq reactions during the TET2 step was generally prolonged to 90 min in order to increase discrimination of protected versus non-protected nucleotides. In order to further enhance TET2-based oxidation protection of modified bases, TET2 concentration was also experimentally increased to double amount in some test reactions, in particular with regard to the methylation detection on m^4^C motifs; this resulted in no perceptible changes in average conversion detection for all of our three main target motifs. Hence, the recommended protocol with standard TET2 enzyme amounts was kept for all following library preparations. To achieve a complete deamination of non-protected cytosines in the next step of sample preparation, samples were generally incubated with 1.5 µl of APOBEC instead of the recommended 1 µl, at 37 °C, and incubation time in this step was increased to 4 h instead of the recommended 3 h.

#### Illumina sequencing specifications of EM-Seq libraries

EM-Seq libraries were sequenced on an Illumina MiSeq instrument using 600 cycles with MiSeq V3 kits (2 × 300 bp paired-end), with at least 20- to 50-fold genome coverage (see below and results for definition of quality standards). Demultiplexing using EM-Seq adaptors was directly performed on the MiSeq instrument. Additional quality controls post-run were performed using FastQC v0.12.0 (http://www.bioinformatics.babraham.ac.uk/projects/fastqc). Trim Galore v0.6.10 (Babraham Institute), a wrapper around Cutadapt (10.14806/ej.17.1.200, was used for quality filtering and Illumina adapter trimming using the following settings: -q20 –length 50 –clip_R1 10 –clip_r2 10.

#### Analysis of the cytosine conversion frequency in EM-Seq data

The bisulfite-converted DNA sequence aligner Bismark v0.22.3 [61] was used to align EM-Seq-derived reads to the reference *H. pylori* genomes of strains N6 [34] or 26695 [35], with default settings. Additionally, reads were also aligned to the pUC19 plasmid and the Lambda phage controls to determine conversion efficiency. Briefly, Bismark detects cytosine- to -thymine transitions resulting from the enzymatic conversion (by APOBEC) of unmethylated cytosines. The ratio of thymine to cytosine at each single-base position is then calculated to determine the conversion frequency for each cytosine in a respective methylatable motif in the genome. To be included in downstream analysis, each sample was required to have a genome coverage above 30 × as well as a conversion frequency for the methylated pUC19-positive control (spike-in) and non-methylated Lambda phage-negative control (spike-in) below 5% and above 95%, respectively. We tested the minimal read depth of the converted genomes to reach greater than 95% overall cytosine coverage, using a *H. pylori* N6 wild type sample with a high coverage of > 50. For this purpose, we randomly downsampled all sample reads to different read depths/coverage of 50-fold, 25-fold, tenfold, and five-fold, respectively, and subjected the remaining reads to the subsequent mapping and downstream conversion analyses as described above. We found that a read depth equal or above 20-fold reproducibly detected more than 95% of all cytosines (converted and non-converted, of genome and both spike-in controls). Furthermore, only cytosines with a minimum isolated read depth of 5 were included in the downstream analysis. All analyzed motifs were detected in the *H. pylori* genome using the Biostrings v2.18.4 Bioconductor R package (https://bioconductor.org/packages/Biostrings). Conversion frequencies [%] from 0 to 100%, *x*-axis, for all single motifs (summarized as motif counts of the same frequency bin on the *y*-axis) in individual samples are represented by bar graph histograms (bins = 15). Single-base resolution of methylation was depicted as dot plots (*x*-axis: genomic location of nucleotide, *y*-axis: conversion frequency (average for each site) in [%]). Comparison of multiple samples with technical replicates are represented by box plot with (i) the median indicated by the central line across the box, (ii) the lower and upper hinges representing the 25th and 75th percentile, respectively, and (iii) the ends of the lower and upper whiskers representing the minimum and maximum data points, respectively. Both types of plots were created with the ggplot2 R package (github.com/tidyverse/ggplot1). To perform the comparison of conversion frequencies between the *H. pylori* strains N6 and 26695, the genomes of both strains were aligned using progressiveMauve v2.4.0 [62] to their respective reference genomes, and the aligned coordinates were extracted from the XMFA output using an in-house R script, and converted into csv format tables. For downstream analysis and between-strain comparisons, the genome coordinates of strain 26695 (NCBI PRJNA175543) were then converted to N6 (NCBI PRJEA89447) genome coordinates. See Additional file 1: Fig. S1C for flow chart of bioinformatic analysis downstream of the Illumina raw reads. The project sequencing data (single read archives) are deposited under project number PRJNA1107780 at the NCBI. Cytosine motif density over the whole genome for all three C-methylated motifs was calculated using the DistAMo webtool (Additional file 1: Fig. S7). 

#### EM-Seq statistics

Differential methylation analysis was performed by modelling the conversion frequencies using logistic-regression and testing the differences between samples with a likelihood ratio chi-square test, as implemented in the MethylKit v0.99.2 R package [63]. Basic overdispersion correction was performed by applying a scaling parameter to the variance estimated by the model (10.1007/978-1-4899-3242-6). Effect size was calculated using read coverage as weights for mean conversion frequencies. The comparison of mean conversion frequencies between different genetic contexts for each motif was performed using Welch’s *t*-test. The project sequencing data (single read archives) are deposited under project number PRJNA1107780 at the NCBI [64]. Conversion frequencies for all experimental EM-Seq datasets are contained in Additional file 3: Table S2.

#### Cluster analysis of EM-Seq data

To identify cytosines with a pattern of lower methylation across the genome, four *H. pylori* N6 wild type replicates (standard growth conditions) were analyzed using K-means clustering (Fig. S5). The optimal number of clusters for each motif was determined using a Scree plot (*n* = 5 for GCGC and *n* = 6 for CCTC and TCTTC), and the resulting clusters were visualized as a heatmap. The conversion frequencies corresponding to the cluster with the lowest average methylation are available in Additional file 4: Table S3.

### Oxford Nanopore Technology (ONT) sequencing and analysis of ONT data

*H. pylori* strain N6 (wild type) was sequenced using the Oxford Nanopore Technology (ONT). One microgram of *H. pylori* N6 genomic DNA, isolated using the DNeasy® UltraClean® Microbial Kit (Qiagen, Hilden, Germany), was used for preparation of libraries using the ONT Ligation Sequencing Kit V14 (SQK-LSK114). All steps were performed as per the manufacturer’s instructions. Twenty femtomole of the final prepared library was loaded on a R10.4.1 MinION flowcell on a MinION Mk1B instrument. The resulting fast5 files were converted to pod5 format using ONT’s converter. Basecalling was performed using the Dorado basecaller in combination with the high accuracy SUP basecalling model and the all context ^m5^C modified bases models. Basecalled reads were aligned to the *H. pylori* N6 strain genome reference sequence using minimap2 with the map-ont presets (REF: 10.1093/bioinformatics/bty191) as integrated within Dorado. Methylation average was calculated as the modified base to read-depth ratio for each cytosine within GCGC motifs. ONT sequencing data are publicly available in the NCBI GenBank repositories under PRJNA1107780 [64].

### Supplementary Information

**Additional file 1:** **Supplementary Fig. S1.** Graphic charts of EM-Seq method and genome-wide methylation at three different cytosine motifs by EM-Seq in *H. pylori* 26695. **Fig. S2.** Cluster analysis with heatmaps of genome-wide cytosine methylation at a single-base quantitative level (EM-Seq) for three different methylated cytosine motifs in *H. pylori* N6. **Fig. S3.** Genome-wide average of single-base GCGC and TCTTC methylation in *H. pylori* N6 and isogenic *luxS* mutant and qPCR of the respective MTase genes. **Fig. S4.** Genome-wide arrangement of local single and overlapping cytosine methylation motifs in *H. pylori* strain N6. **Fig. S5.** Analysis of overlapping GCGC motif doublets in *H. pylori* N6 by EM-Seq and ONT Sequencing. **Fig. S6.** Local quantitative analysis of methylation in *H. pylori* genes regulated by MTase M.HpyAVIII (GCGC) using EM-Seq. **Fig. S7.** DistAMo analysis of genome-wide localization and distribution of cytosine MTase target motifs in *H. pylori*

**Additional file 2:** **Table S1.** Genome annotations, nucleotide positions and locus tags of draft genome of *H. pylori* N6 (NCBI PRJEA89447).

**Additional file 3:** **Table S2.** EM-seq conversion frequencies [%] for all cytosine-methylated sites (G^m5^CGC, T^m4^CTTC, ^m5^CCTC motifs) in the *H. pylori* N6 genome.

**Additional file 4:** **Table S3.** Members of gene cluster of consistently lower methylated sites in *H. pylori* N6 by EM-Seq analysis.

**Additional file 5:** **Table S4.** Differentially methylated sites in the *H. pylori N6* genome under conditions of methionine depleted/replete conditions**.**

## Data Availability

All data generated or analyzed during this study are included in this published article, its supplementary information files (Supplementary Tables in Additional files 2, 3, 4, 5) and publicly available repositories (NCBI) as stated in the “ Methods” section. Raw sequencing data supporting the conclusions of this article are available in the SRA database (accession ID PRJNA1107780, BioSample accession IDs SAMN41247928, SAMN41247929, SAMN41247930, SAMN41247931, SAMN41247932, SAMN41247933, SAMN41247934, SAMN41247935, SAMN41247936, SAMN41247937, SAMN41247938, SAMN41247939, SAMN41247940, SAMN41247941, SAMN41247942, SAMN41247943, SAMN41247944, SAMN41247945, SAMN41247946, SAMN41247947, SAMN41247948), which are accessible with the following link: https://www.ncbi.nlm.nih.gov/sra/?term=PRJNA1107780. Additional datasets and materials of the study will be provided by the corresponding author on reasonable request.

## Abbreviations

APOBEC: Apolipoprotein B mRNA editing enzyme catalytic polypeptide
CDS: Coding sequence
ELISA: Enzyme-linked immunosorbent assay
EM-Seq: Enzymatic methyl sequencing
LPS: Lipopolysaccharides
MTAN: Methylthio-adenosine nucleosidase
MTase: Methyltransferase
ONT: Oxford Nanopore Technology
RIMS-Seq: Rapid Identification of Methylase Specificity Sequencing
SAH: S-adenosyl-homocysteine
SAM: S-adenosyl methionine (AdoMet)
SEM-Seq: Single-Enzyme 5-Methylctyosine Sequencing
SMRT: Single Molecule, Real-Time
SRH: S-ribosyl homocysteine
TET2: Ten-Eleven Translocation methylcytosine dioxygenase 2
