# Supplementary material for: Single-base resolution quantitative genome methylation analysis in the model bacterium Helicobacter pylori by enzymatic methyl sequencing (EM-Seq) reveals influence of strain, growth phase, and methyl homeostasis
Source: BMC Biol. 2024 May 29;22:125. doi: 10.1186/s12915-024-01921-1 (PMC11134628; doi:10.1186/s12915-024-01921-1)
Supplement: Supplementary file 1 — Additional file 1: Supplementary Fig. S1. Graphic charts of EM-Seq method and genome-wide methylation at three different cytosine motifs by EM-Seq in H. pylori 26695. Fig. S2. Cluster analysis with heatmaps of genome-wide cytosine methylation at a single-base quantitative level (EM-Seq) for three different methylated cytosine motifs in H. pylori N6. Fig. S3. Genome-wide average of single-base GCGC and TCTTC methylation in H. pylori N6 and isogenic luxS mutant and qPCR of the respective MTase genes. Fig. S4. Genome-wide arrangement of local single and overlapping cytosine methylation motifs in H. pylori strain N6. Fig. S5. Analysis of overlapping GCGC motif doublets in H. pylori N6 by EM-Seq and ONT Sequencing. Fig. S6. Local quantitative analysis of methylation in H. pylori genes regulated by MTase M.HpyAVIII (GCGC) using EM-Seq. Fig. S7. DistAMo analysis of genome-wide localization and distribution of cytosine MTase target motifs in H. pylori [file 12915_2024_1921_MOESM1_ESM.pdf]

# Supplementary Figures

## Single-base resolution quantitative genome methylation analysis in the model bacterium Helicobacter pylori by enzymatic methyl sequencing (EM-Seq) reveals influence of strain, growth phase and methyl homeostasis

Lubna Patel[§], Florent Ailloud[§], Sebastian Suerbaum, Christine Josenhans*

Max von Pettenkofer Institute, Chair for Medical Microbiology, Faculty of Medicine, LMU Munich, Pettenkoferstr. 9a, 80336 Munich; DZIF site Munich

[§] contributed equally

* address correspondence to: josenhans@mvp.lmu.de

# Figure S1

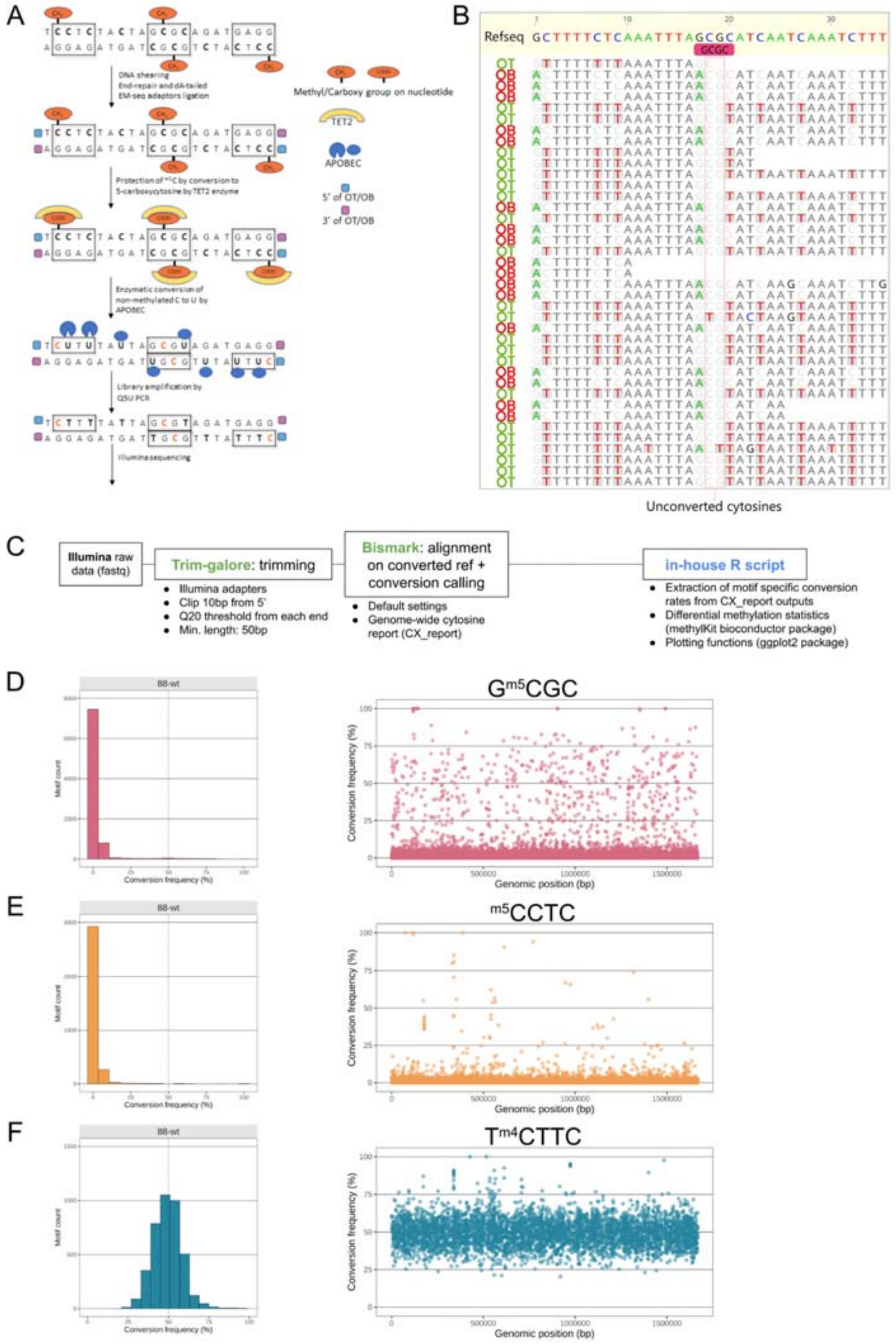


**Figure S1. Graphic charts of EM-Seq method and genome-wide methylation at three different cytosine motifs by EM-Seq in *H. pylori* 26695 on a global and local scale. A.** shows a graphic chart of the main reaction steps of the EM-Seq method. The schematic is derived from method description by manufacturer New England Biolabs (NEB) and in Vaisvila R. et al., 2021; PMID:34140313. **B.** depicts read alignment (> 20-fold coverage) of converted and non-converted cytosines around a G$^{m5}$CGC motif (red box), with the reference sequence (RefSeq; *H. pylori* N6, upper line) as used for the final EM-Seq analysis. OT (original top, green) are the sense strand sequences from the EM-Seq library; OB (original bottom, red) are the antisense strand sequences from the same pipeline (original guanosine nucleotides are not converted). Reverse sequence reads have been reverse-complement converted for/during the alignment. Blue cytosines (C) in the sense strand indicate potentially convertible C bases. While most OT Cs are converted, since they are not methylated, Cs in the GCGC motif (indicated in the red box) are predominantly not converted, indicating their methylation. **C.** Flow chart of bioinformatic analysis pipeline of EM-Seq data. **D**, **E**, **F**, DNA conversion frequency in [%] (direct inverse measure of methylation) of three different methylated cytosine motifs in *H. pylori* wild type 26695 (synonymous name: 88-wt [replicate R1, Main Table 1]). **D**. G$^{m5}$CGC motif, **E**. $^{m5}$CCTC motif, **F.** T$^{m4}$CTTC motif. Bar graphs (left panels, y-axis shows motif counts, x-axis shows conversion frequency in bins of 50) depict the conversion frequency per genomic motifs, while distribution and conversion average of local single motif bases along the genome length (x-axis) are shown as dot plots (right panels). Each motif cytosine is visualized as one colored dot. The three cytosine motifs vary in average percentages of conversion (y-axis, 0 % conversion corresponds to 100% methylation). Vertical line in left-hand panels denotes 50% conversion. Conversion data for D, E, F in Additional File 3: Table S2.

# Figure S2

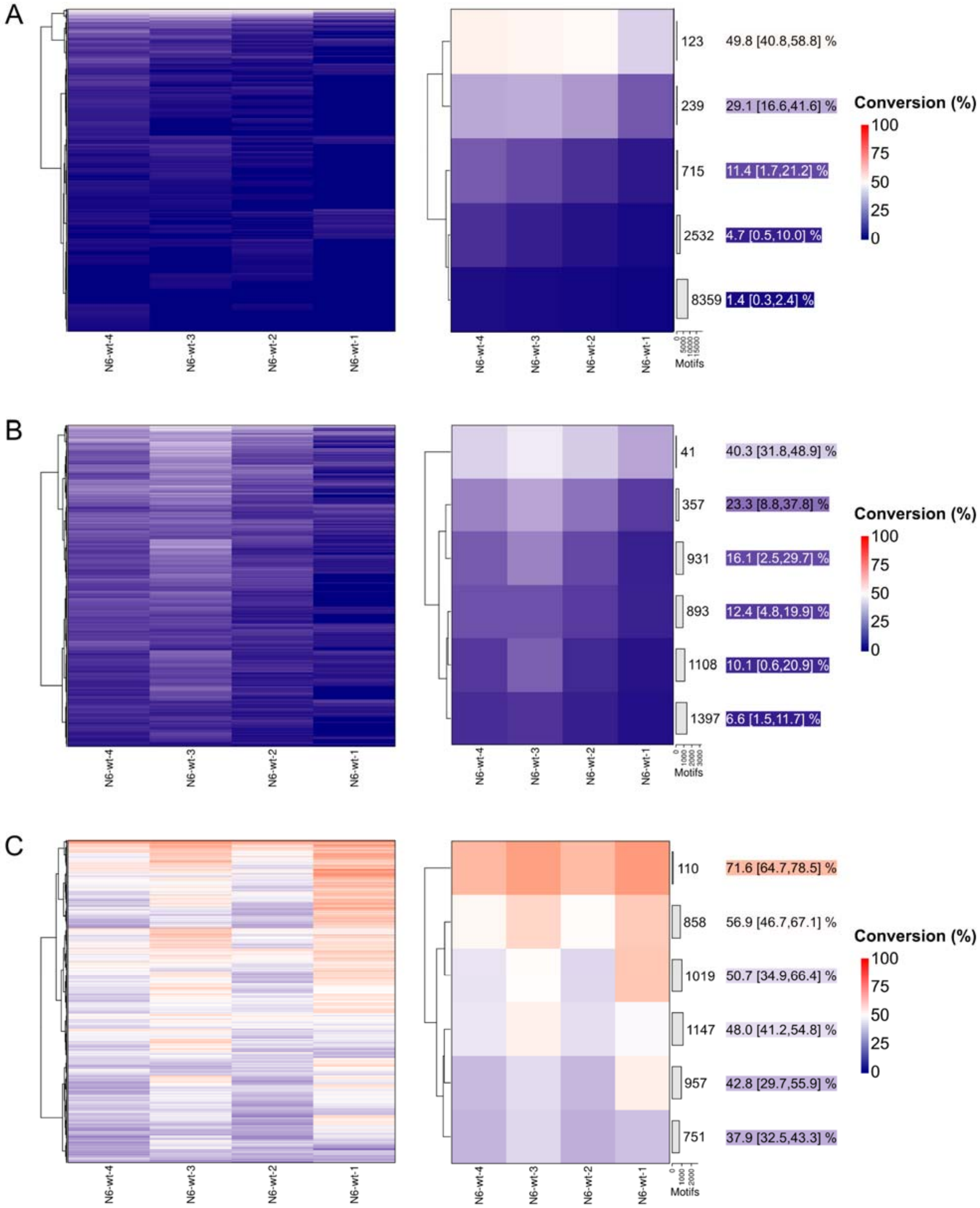


**Figure S2. Cluster analysis with heatmaps of genome-wide cytosine methylation at a single-base quantitative level in *H. pylori* detected by EM-Seq. Three different methylated cytosine motifs are analyzed separately. A.** GCGC motifs; **B.** CCTC motifs; **C.** TCTTC motifs. Four biological replicates of *H. pylori* N6 wild type strain grown under standard culture conditions are depicted (Table 1). Single motif methylation is depicted with the direct inverse measure of motif conversion [%] as detected by EM-Seq. Left panels for each motif show all detected clusters, and right panels depict a collapsed view of only five (GCGC) or six (CCTC and TCTTC) clusters (cluster definition see methods), distinguished by differential quantitative single-nucleotide methylation. Number of motifs and average methylation (with 95% confidence intervals) of the respective sites included in each cluster in the right panels are indicated to the right close to the bar graphs. The respective upper cluster of significantly lower methylated local cytosines was further analyzed for gene context. The genes and genomic location of sites in upper cluster (consistently low methylated) are summarized in Additional File 4: Table S3 (Table S3 only lists methylated bases in CDS, not at intergenic sites). Full conversion data for the four replicates in Additional File 3: Table S2.

# Figure S3

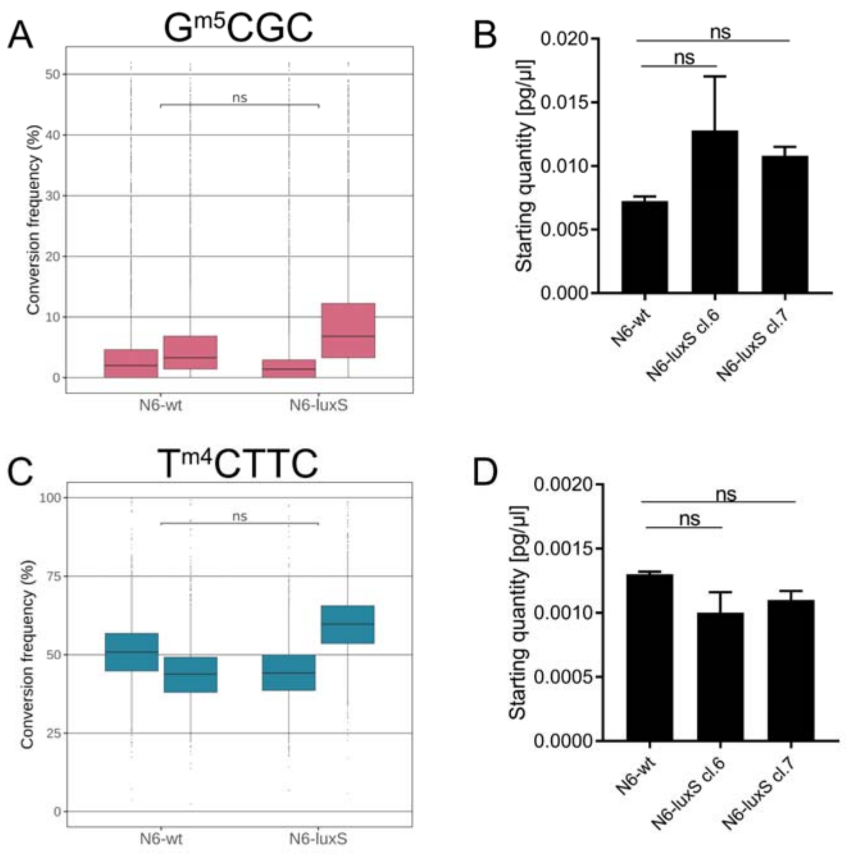


**Figure S3. Genome-wide average single-base DNA methylation of cytosine motifs in *H. pylori* wild type (N6-wt) compared to isogenic mutant in *luxS* (N6-luxS) in liquid medium and qPCR analyses of respective MTase genes. A.** and **C.,** genome-wide average of cytosine methylation (shown as EM-Seq conversion frequency [%]) in GCGC (A) or TCTTC (C) motif methylation; two biological and technical replicates each (R1 [N6-wt3][N6-luxS-R1] and R2 [N6-wt4][N6-luxS-R2], Table 1) are summarized as box-plots with whiskers. Chi-square p-values for significant differences between strains: n.s. = non-significant. Conversion data are summarized in Additional file 3: Table S2. **B.** Transcript quantification (qPCR) of gene HP1121 ($G^{m5}$CGC MTase, M.HpyAVIII) in *H. pylori N6* wild type strain in comparison with its isogenic *luxS* mutant. Two independent mutant clones (cl.6 and cl.7) are shown, and three biological replicates for each condition are summarized in each bar. **C**. TCTTC motif. Chi-square p-values: n.s. is non-significant. **D.** Transcript quantification (qPCR) of gene HP1368 ($T^{m4}$CTTC MTase, M2.HpyAII) in *H. pylori* N6 wild type strain in comparison with its isogenic *luxS* mutant. Two independent mutant clones (cl.6 and cl.7) are shown, and three biological replicates quantitated for each condition are summarized in each bar. qPCR reactions of each replicate were performed in technical triplicates, standardized for specific transcript amounts [pg/µl] using internal standards for each specific gene, and normalized against 16S rRNA transcript for each replicate. Statistical significances of differences of qPCR results between conditions were calculated using one-way ANOVA. Significances are marked as: ns = non-significant. While there is a trend for the *luxS* mutant clones to have higher and lower transcript amounts for the two MTase genes, respectively, this was not significant.

# Figure S4

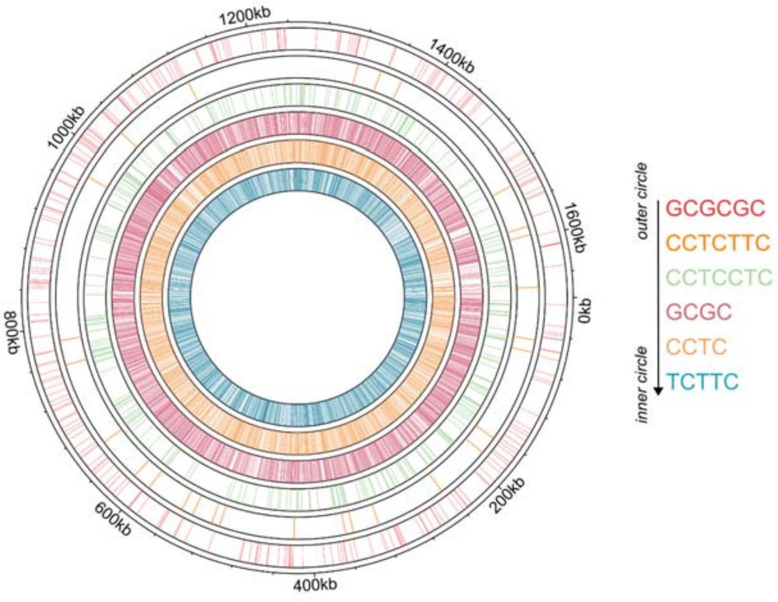


**Figure S4. Genome-wide arrangement of local single and overlapping cytosine methylation motifs in *H. pylori* (strain N6).** The colored genome circles depict the locations of all single (inner circles) and overlapping (outer circles) cytosines in methylated motifs, as indicated in the legend to the right.

# Figure S5

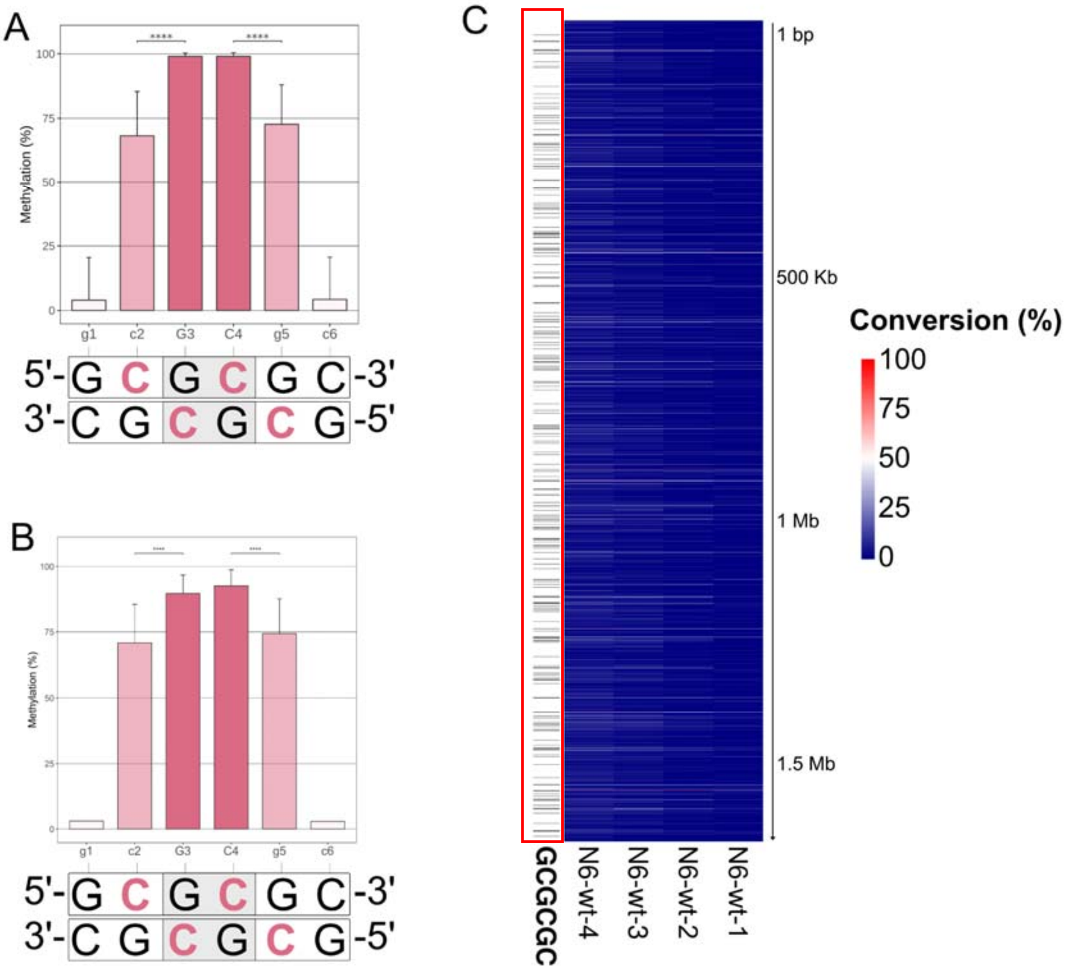


**Figure S5. Analysis of overlapping GCGC motif doublets in *H. pylori* N6 wild type which have partially reduced genome-wide cytosine methylation as detected in EM-Seq and Oxford Nanopore technologies (ONT) sequencing, with local genomic distribution.**

**A.** Left panel demonstrates the EM-Seq methylation average for the central versus external methylated cytosines in the overlapping GCGC motif doublets for N6 wild type strain (summarized from four replicates of wt N6, Table 1), corresponding to main Fig. 7. Full conversion data in Additional file 3: Table S2.

**B.** Analysis of reduced single nucleotide methylation in overlapping GCGC motifs by Oxford Nanopore sequencing. *H. pylori* N6 wild type bacteria cultured under standard growth conditions were sequenced by ONT and further analyzed (methods). A similar preference (statistically significant difference) for lower average of local methylation in the external motif cytosines (c/g2 and g/c5) as with the EM-Seq methodology was quantitated. EM-Seq showed a slightly higher accuracy and sensitivity for methylation detection than ONT (164-fold coverage). Blue-colored matrix (heatmap) in **C.**, derived from four biological replicates of *H. pylori* N6 wild type strain grown under similar standard culture conditions (main Table 1, same samples as in Fig. S6), shows the distribution and methylation average (shown as conversion [%] as a direct inverse measure of methylation) of each overlapping GCGC motif throughout the genome (y-axis, bordered in red, shows genome positions from top to bottom; each GCGC motif is marked by a blueish line). Genome-wide distribution of overlapping GCGC motifs along the genome seems to be distributed rather evenly, with a 6.5-fold preference of such motifs to be located in coding regions over upstream/promoter regions (own unpublished data). All sequencing data are deposited in NCBI GenBank under project accession number PRJNA1107780.

# Figure S6

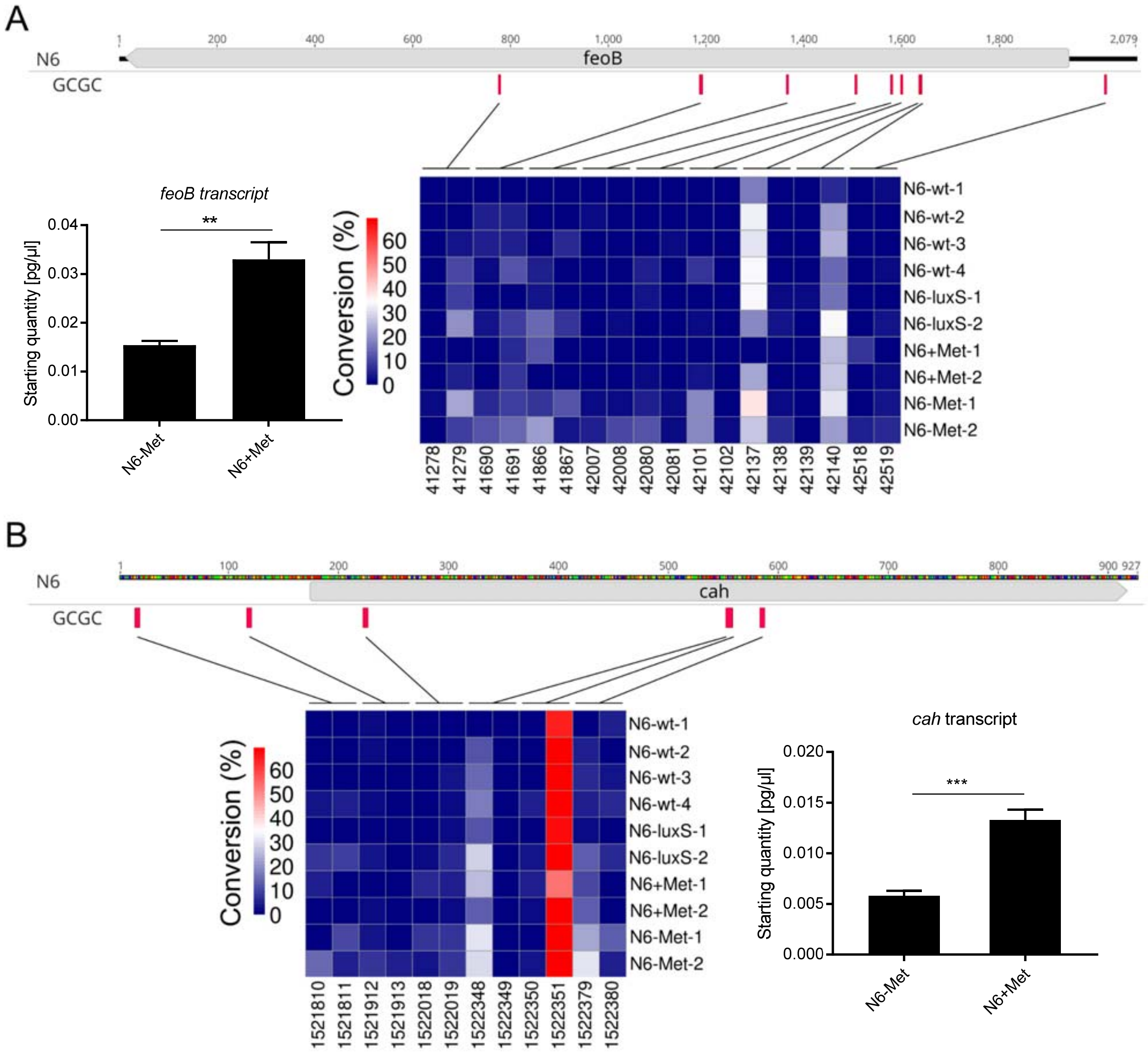


**Figure S6. Local quantitative analysis of methylation in *H. pylori* genes regulated by MTase M.HpyAVIII (GCGC) using EM-Seq. A.** Analysis of local quantitative GCGC motif methylation (heatmaps, showing EM-Seq data as conversion [%], of five different experimental conditions of *H. pylori* strain N6 in biological replicates, Table 1; Additional file 3: Table S2, in iron transporter subunit *feoB* gene including its upstream promoter region (Estibariz I. et al., 2019; PMID: 30624738). **B.** Local site-specific quantification of GCGC motif methylation (heatmaps as in A) in the *H. pylori* alpha-carbonic anhydrase gene *cah*, including its upstream promoter region. In both examples, in particular the motif overlaps (e.g. *cah* site 1522351, *feoB* site 42137) consistently produce quantitatively lower single-site methylation on cytosine bases. Alongside, *feoB* (A) and *cah* (B) transcripts were quantitated by qPCR under methionine-low and methionine-supplemented conditions (methods), and are significantly different (decreased) under methionine-low conditions. The significantly differential methylation of *feoB* and *cah*-located cytosine sites (e.g. *cah* site 1522380, *feoB* site 42101; Additional file 2: Table S1) under low-methionine conditions is shown in Additional file 5: Table S4. *cah* also has a consistently low-methylated site (red), as also listed in Additional file 4: Table S3. qPCR results were standardized for transcript amounts (Starting Quantity [pg/µl]) to internal specific gene controls and normalized to 16S transcript for each condition. The qPCR reactions were performed in technical triplicates. Significances (student's *t*-test) are marked as: * p < 0.05; ** p < 0.01; **** p < 0.0001; ns = non-significant.

## Figure S7

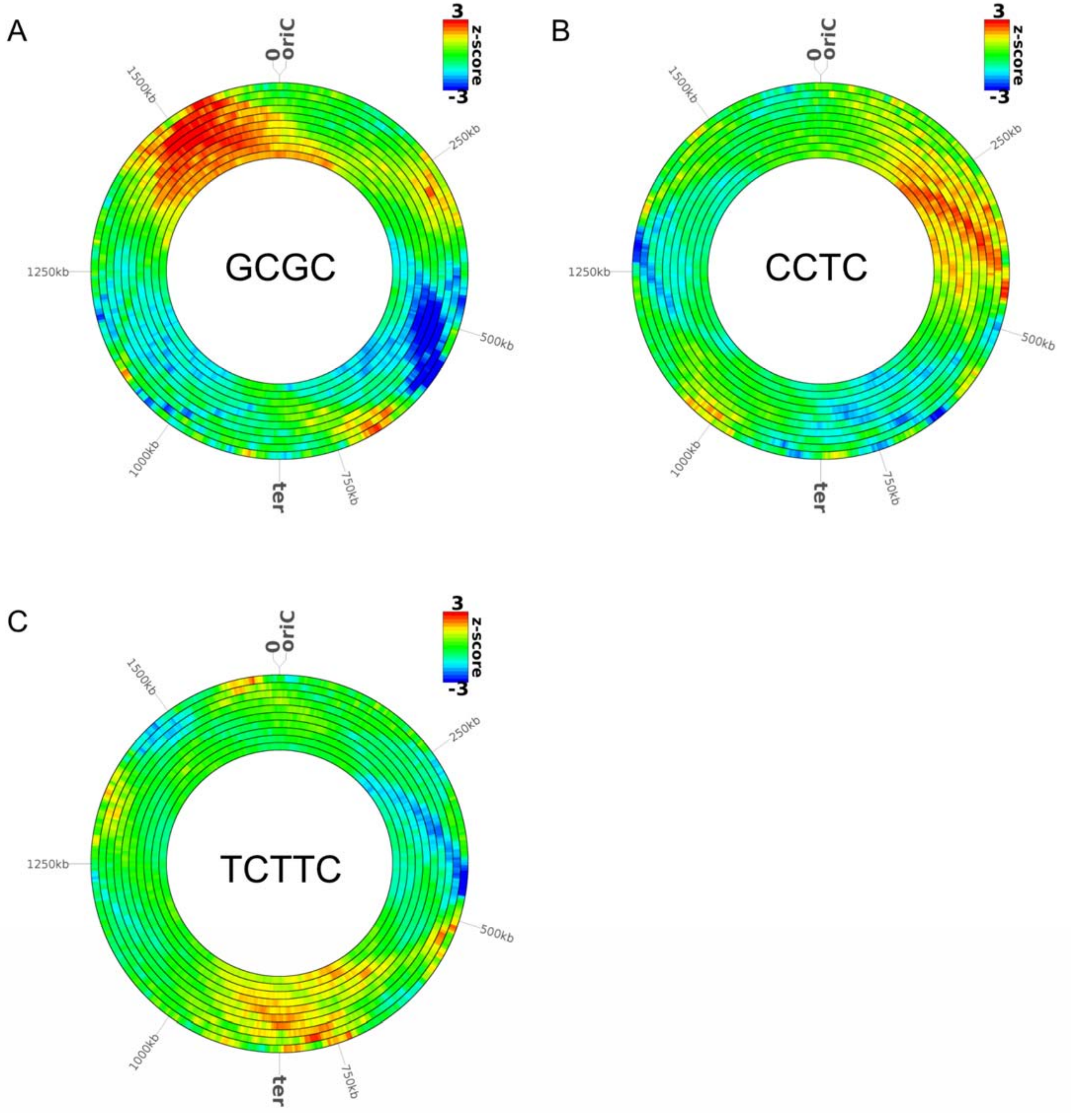


**Figure S7. DistAMo analysis of genome-wide localization and distribution of cytosine MTase target motifs in *H. pylori*.** Analysis of motif distribution along the genome of the *H. pylori* 26695 strain (NCBI PRJNA175543) is shown.
The analysis was performed using the DistAMo algorithm (https://www.computational.bio.uni-Giessen.de/distamo/; https://doi.org/10.3389/fmicb.2016.00283); Each concentric colored ring shows the motif distribution across different window sizes, ranging from 50 kb for the outer ring, to 500 kb for the inner ring, increasing by steps of 50 kb. Over- and under-representation of a given motif are indicated by a Z-score above 2 (reddish coloring) and below -2 (blueish coloring), respectively. The *cag* pathogencity island in strain 26695 is located between ~547kb and ~583kb, and this ca. 32 kb region seems to be depleted in GCGC motifs in comparison to the rest of the genome. **A.** GCGC motif distribution analysis; **B.** CCTC motif distribution analysis; **C.** TCTTC motif distribution analysis.
